# Computer-Aided Design of Orally Bioavailable Pyrrolidine Carboxamide Inhibitors of Enoyl-Acyl Carrier Protein Reductase of *Mycobacterium tuberculosis* with Favorable Pharmacokinetic Profiles

**DOI:** 10.3390/ijms161226196

**Published:** 2015-12-12

**Authors:** Affiba Florance Kouassi, Mawa Kone, Melalie Keita, Akori Esmel, Eugene Megnassan, Yao Thomas N’Guessan, Vladimir Frecer, Stanislav Miertus

**Affiliations:** 1 Laboratoire de Physique Fondamentale et Appliquée, University of Abobo Adjamé—Nangui Abrogoua, Autoroute d’Abobo, Abidjan 02, Cote D’Ivoire; oreli060@yahoo.fr (A.F.K.); keitamelalie@yahoo.fr (M.K.); elvicee@yahoo.fr (A.E.); 2 Laboratoire de Chimie Organique et des Substances Naturelles, University of Cocody—Felix Houphouët-Boigny, Avenue de l’Université, Abidjan 22, Cote D’Ivoire; kone_m2001@yahoo.fr (M.K.); nguessanyat@yahoo.fr (Y.T.N.); 3 International Centre for Science and High Technology, UNIDO, Area Science Park, Trieste I-34012, Italy; 4 Faculty of Pharmacy, Comenius University in Bratislava, Bratislava SK-83232, Slovakia; frecer@fpharm.uniba.sk; 5 International Centre for Applied Research and Sustainable Technology, Bratislava SK-84104, Slovakia; stanislav.miertus@icarst.org; 6 Faculty of Natural Sciences, University of SS. Cyril and Methodius, Trnava SK-91701, Slovakia

**Keywords:** tuberculosis, pyrrolidine carboxamides, InhA inhibitors, molecular modeling, QSAR model, pharmacophore model, virtual combinatorial library, *in silico* screening, ADME properties

## Abstract

We have carried out a computational structure-based design of new potent pyrrolidine carboxamide (PCAMs) inhibitors of enoyl-acyl carrier protein reductase (InhA) of *Mycobacterium tuberculosis* (*MTb*). Three-dimensional (3D) models of InhA-PCAMx complexes were prepared by *in situ* modification of the crystal structure of InhA-PCAM1 (Protein Data Bank (PDB) entry code: 4U0J), the reference compound of a training set of 20 PCAMs with known experimental inhibitory potencies (*IC*_50_^exp^). First, we built a gas phase quantitative structure-activity relationships (QSAR) model, linearly correlating the computed enthalpy of the InhA-PCAM complex formation and the *IC*_50_^exp^. Further, taking into account the solvent effect and loss of inhibitor entropy upon enzyme binding led to a QSAR model with a superior linear correlation between computed Gibbs free energies (ΔΔ*G*_com_) of InhA-PCAM complex formation and *IC*_50_^exp^ (p*IC*_50_^exp^ = −0.1552·ΔΔ*G*_com_ + 5.0448, *R*^2^ = 0.94), which was further validated with a 3D-QSAR pharmacophore model generation (PH4). Structural information from the models guided us in designing of a virtual combinatorial library (VL) of more than 17 million PCAMs. The VL was adsorption, distribution, metabolism and excretion (ADME) focused and reduced down to 1.6 million drug like orally bioavailable analogues and PH4 *in silico* screened to identify new potent PCAMs with predicted *IC*_50_^pre^ reaching up to 5 nM. Combining molecular modeling and PH4 *in silico* screening of the VL resulted in the proposed novel potent antituberculotic agent candidates with favorable pharmacokinetic profiles.

## 1. Introduction

**Figure 1:**
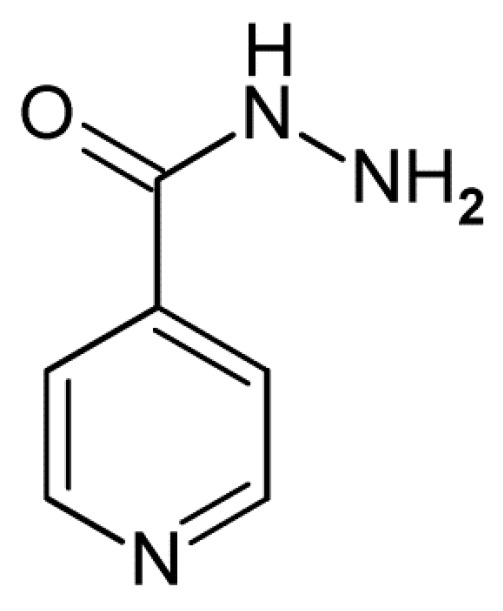
Chemical structure of antituberculotic agent—prodrug isoniazid (INH).

The World Health Organization (WHO) has set as its next goal the decrease of incidence of tuberculosis (TB) and TB-related deaths by more than 90% in the coming 20 years [1]. However, due to emerging and rapidly spreading multi-drug resistant and extensively-drug resistant strains of *Mycobacterium tuberculosis* (*MTb*), the causative agent of TB, this goal will be difficult to achieve without the discovery of new potent antituberculotic drugs. Despite the increasing worldwide incidence of TB and the threat for the public health, no novel antituberculotic drugs have been introduced into clinical practice over the past decades. It is therefore imperative to diversify the mycobacterial drug targets in order to fight the increasing incidence of drug-resistant strains of *MTb*. The oxidoreductase activity of enoyl-acyl carrier protein reductase (InhA or ENR) plays a key role in the type-II fatty-acid synthesis (FAS-II system) of *MTb*. This essential enzyme catalyzes the elongation cycle of biosynthesis of mycolic acid, which is a vital component of the mycobacterial cell wall [2]. Thus, InhA represents a validated drug target of antituberculotic agents and has been indicated in the Special Programme for Research & Training in Tropical Diseases of WHO (TDR) targets database as an attractive pharmacological target for design of new drug candidates [3,4]. In addition, the InhA sequence and structural organization for bacterial species are distinctly different from mammalian’s fatty acid biosynthesis enzymes. Isoniazid prodrug (INH), Figure 1, is a first-line medication used for prevention and treatment of TB. The INH is a prodrug that must be activated by the bacterial catalase-peroxidase enzyme (KatG) which couples the isonicotinic acyl with the reduced form of nicotinamide adenine dinucleotide (NADH) to form an isonicotinic acyl-NADH complex. The complex binds tightly to InhA, blocks the natural substrate and hinders the action of fatty acid synthase, which hampers the synthesis of mycolic acid [5].

The observed resistance of *MTb* to INH arises from mutations in the mycobacterial KatG and its reduced ability to activate the prodrug [6,7,8].

**Figure 2:**
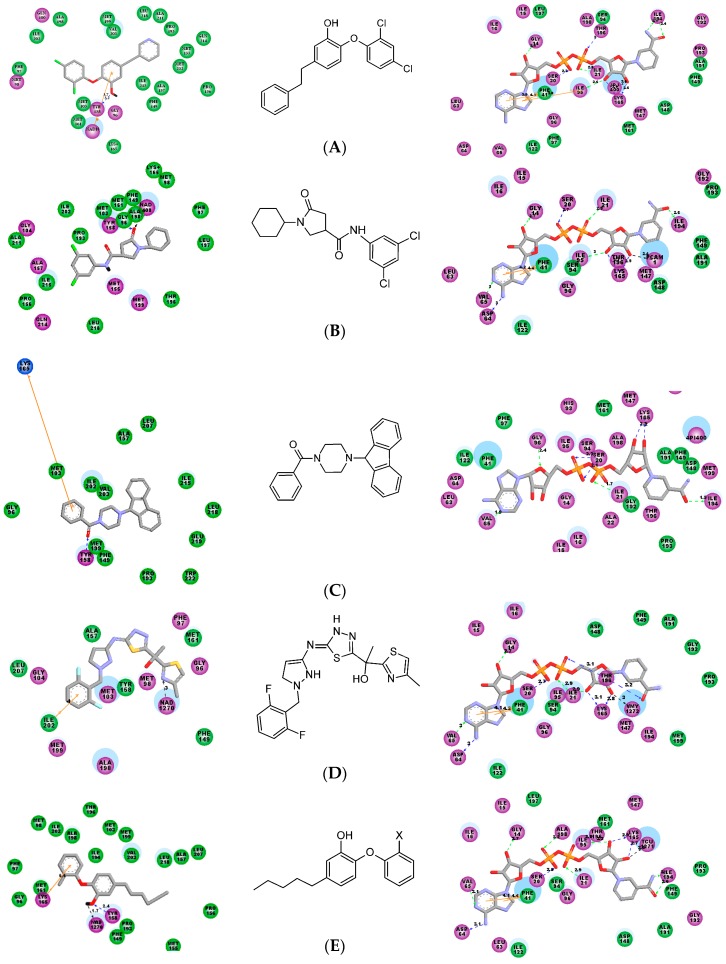
(**Left**) 2D interaction diagram of designed nanomolar enoyl-acyl carrier protein reductase (InhA) inhibitors. Middle: most important affinity features: van der Waals, σ–π, π–π, cation–π (orange line), hydrogen bonds (HBs) (black, blue and green dashed line) and hydrophobic; (**Right**) pertinent nicotinamide adenine dinucleotide (NADH) conformation. (**A**) Triclosan (*IC*_50_ = 21 nM), HB: Tyr158, π–π: NAD, PDB: 3FNH [9]; (**B**) Pyrrolidine carboxamide (*IC*_50_ = 390 nM), HB: Tyr158 and NAD, PDB: 4U0J [12]; (**C**) Aryl amides (*IC*_50_ = 90 nM), cation–π: Lys165, 2NSD, *in situ* modified [13]; (**D**) Methyl Thiazole (*K*_d_ = 13 nM), HB: NADH, σ–π: Ile202, 4BPQ [1]; (**E**) Alkyl diphenyl ether, (*K*_i_ = 22 pM), HB: Tyr158, NADH, cation–π: Lys165, 2X23 [11]. Active site residues are presented in balls colored by interaction type (green for van der Waals, magenta for polar, charge and hydrogen bond while light blue for water or solvent accessible surface).

**Figure 3:**
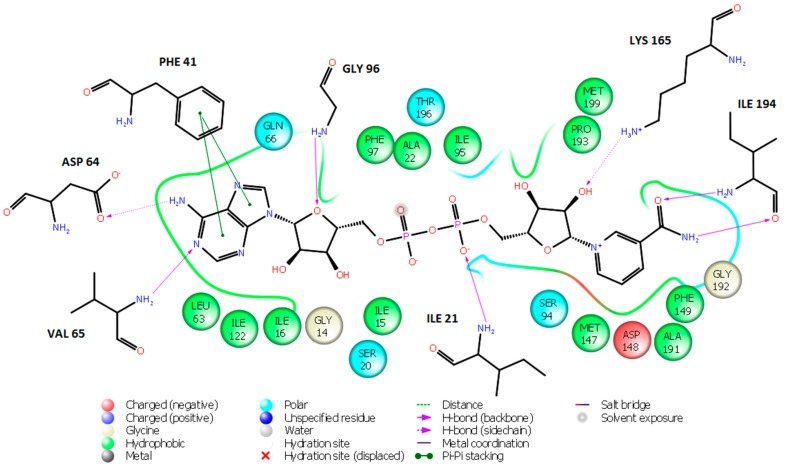
NADH conformation and 2D-depiction of cofactor-InhA interactions in the crystal structure 4DRE.

Several research groups were working on the discovery of InhA inhibitors not requiring KatG activation which were based on various scaffolds: triclosan [9], diphenyl ether [10,11], pyrrolidine carboxamide [12] and arylamide derivatives [13], all displaying intermediate inhibitory potencies. Recently, structural requirements for efficient InhA inhibitors have been evaluated by 2D- and 3D-quantitative structure-activity relationships (QSAR) methods, hologram QSAR (HQSAR) and comparative molecular field analysis (CoMFA) [14]. These studies indicated that a potent InhA inhibitor should be a relatively long molecule, which binds to the InhA next to the NADH cofactor binding site. This inhibitor should also contain a bulky group that selectively fits into a hydrophobic pocket of InhA constituted by residues Met155, Pro193, Ile215, Leu217, Leu218 and Trp222 that is located near to a larger solvent accessible cavity [14]. The crystal structure conformation of the NADH cofactor in InhA complexes with bound nanomolar inhibitors is shown on Figure 2. Except for the aryl amide inhibitor (Figure 2C), conformation of the NADH in these ternary complexes is extended, similar to the NADH conformation in the reference binary InhA-cofactor complex (PDB entry 4DRE [15]), Figure 3.

The best InhA inhibitor designed so far belongs to the pyrrolidine carboxamide family (PCAM) and displays an inhibitory concentration *IC*_50_ of 390 nM [12]. Since PCAMs are direct InhA inhibitors bypassing the drug resistance related to the need for KatG activation, a possibility of optimizing the *IC*_50_ of PCAMs down to one digit nanomolar concentrations, would be rather attractive. Previous SAR knowledge of the existing PCAMs can be summarized as follows: halogen atom substitution in *para*-position of the benzene ring is detrimental for the potency, size limitation for the electron-withdrawing group in *meta*-position is required although that position is preferred to *ortho*. As we can see on Figure 2B, the lack of π–π stacking, cation–π or σ–π interactions explains partially the relatively low potency and opens the gate to the design of more potent analogues of the PCAM family. In this study, we have built a Hansch-type “complexation” QSAR model of InhA inhibition for a training set of known PCAMs inhibitors [12] and correlated computed Gibbs free energies of InhA-PCAMx complex formation with their observed inhibitory potencies. The robustness of this QSAR model was confirmed by a four features 3D-QSAR pharmacophore model (PH4) (see Section 3.8), which was prepared with help of bound conformations of the training set of PCAMs.

Furthermore, we have built a virtual combinatorial library of PCAM analogues with the aim to design more potent orally bioavailable InhA inhibitors. The initial diversity library was focused using computed adsorption, distribution, metabolism and excretion (ADME)-related properties and a subset of predicted orally bioavailable PCAM analogues. In a subsequent screening, generated virtual library of analogues were compared to the PH4 pharmacophore model in order to identify analogues which can fit into the mycobacterial InhA binding site. Preference was given to analogues substituted on the benzene ring so as to enhance the van der Waals interactions in the hydrophobic pocket of the InhA binding site. Based on the complexation QSAR model we were able to predict the activities of the designed PCAM analogues and select the best analogues with the highest predicted inhibitory potencies. Finally, ADME profiles of the best-designed analogues were estimated and compared with those of the antituberculotic drugs currently used in the clinical practice.

## 2. Results and Discussion

A training set of 20 PCAMs and validation set of 3 PCAMs (Table 1) were selected from a homogeneous series of InhA inhibitors for which experimentally determined inhibitory activities were available from a single laboratory [12]. The whole series was obtained by substitutions at five positions of the aromatic ring of 1-cyclohexyl-5-oxo-*N*-phenylpyrrolidine-3-carboxamide (PCAM1) as shown in Table 1. Their experimental inhibitory concentrations *IC*_50_^exp^ [12] cover a concentration range sufficiently wide to serve well for building of a reliable QSAR model of InhA inhibition.

**Table 1:** Training set and validation set of pyrrolidine carboxamide (PCAMx) inhibitors of enoyl-acyl carrier protein reductase (InhA) [12] used in the preparation of quantitative structure-activity relationships (QSAR) model of inhibitor binding.

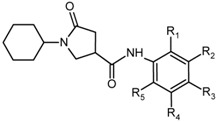						
Training Set	R_1_	R_2_	R_3_	R_4_	R_5_	*IC*_50_^exp^ (μM)
PCAM1	–H	–H	–H	vH	–H	10.66
PCAM2	–H	–H	–Br	–H	–H	28.02
PCAM3	–H	–Br	–H	–H	–H	0.89
PCAM4	–H	–Cl	–H	–H	–H	1.35
PCAM5	–H	–CF_3_	–H	–H	–H	3.51
PCAM6	–H	–H	–CH_2_COOH	–H	–H	73.58
PCAM7	–H	–NO_2_	–H	–H	–H	10.59
PCAM8	–Br	–H	–H	–H	–H	101.00
PCAM9	–H	vCl	–H	–Cl	–H	0.39
PCAM10	–Cl	–H	–Cl	–H	–H	56.02
PCAM11	–Cl	–H	–H	–Cl	–H	56.50
PCAM12	–CH_3_	–Cl	–H	–H	–H	23.12
PCAM13	–H	–Cl	–F	–H	–H	14.83
PCAM14	–H	–F	–F	–H	–H	1.49
PCAM15	–H	–CH_3_	–H	–CH_3_	–H	3.14
PCAM16	–CH_3_	–H	–H	–Cl	–H	0.97
PCAM17	–H	–CF_3_	–H	–CF_3_	–H	3.67
PCAM18	–H	–CH_2_(CH_3_)_2_	–H	–H	–H	5.55
PCAM19	–H	–OCH_3_	–H	–CF_3_	–H	1.30
PCAM20	–H	–Br	–H	–CF_3_	–H	0.85
**Validation Set**	**R_1_**	**R_2_**	**R_3_**	**R_4_**	**R_5_**	***IC*_50_^exp^ (μM)**
PCAM21	–H	–CH_3_	–H	–H	vH	16.79
PCAM22	–H	–H	–Cl	–H	–H	101.00
PCAM23	–H	–CH_3_	–Br	–H	–H	37.41

### 2.1. Quantitative Structure-Activity Relationships (QSAR) Model

The relative Gibbs free energy of the enzyme-inhibitor (E:I) complex formation was computed for the InhA-PCAMx complexes prepared by *in situ* modification of the template inhibitor PCAM1 within the binding site of InhA of the refined crystal structure (PDB entry code 4U0J [12]), as described in the Methods Section. Table 2 lists computed values of Gibbs free energies of complex formation (ΔΔ*G*_com_) and its components, Equation (7) for the training and test sets of pyrrolidine carboxamide inhibitors [12].

**Table 2:** Complexation Gibbs free energy (binding affinity) and its components for the training set of InhA inhibitors PCAM1-20 and validation set inhibitors PCAM21-23.

Training Set ^a^	*M*_W_^b^	ΔΔ*H*_MM_^c^	ΔΔ*G*_sol_^d^	ΔΔ*TS*_vib_^e^	ΔΔ*G*_com_^f^	*IC*_50_^exp^ ^g^
	(g·mol^−1^)	(kcal·mol^−1^)	(kcal·mol^−1^)	(kcal·mol^−1^)	(kcal·mol^−1^)	(μM)
PCAM1	286	0	0	0	0	10.66
PCAM2	365	1.63	−0.49	−2.47	3.61	28.02
PCAM3	365	−6.69	−0.03	−2.09	−4.62	0.89
PCAM4	320	−5.14	−0.25	−1.77	−3.62	1.35
PCAM5	354	−4.55	−1.81	−3.90	−2.46	3.51
PCAM6	328	1.60	0.12	−0.01	1.73	73.58
PCAM7	331	−3.25	−0.33	−2.30	−1.28	10.59
PCAM8	365	3.45	−0.38	−2.41	5.48	101.00
PCAM9	355	−7.51	−0.57	0.18	−8.25	0.39
PCAM10	355	1.25	−0.36	−2.83	3.72	56.02
PCAM11	355	6.15	−0.52	0.13	5.50	56.50
PCAM12	335	1.66	−0.43	0.21	1.02	23.12
PCAM13	339	−3.11	0.11	−2.92	−0.09	14.83
PCAM14	322	−4.91	−0.30	0.12	−5.34	1.49
PCAM15	314	−4.68	−0.25	−0.69	−4.24	3.14
PCAM16	335	−3.54	−0.42	1.42	−5.38	0.97
PCAM17	423	−8.87	−0.13	−6.92	−2.07	3.67
PCAM18	328	1.11	−1.37	3.11	−3.36	5.55
PCAM19	385	−7.71	−0.70	−4.68	−3.74	1.30
PCAM20	433	−9.56	−0.80	−3.70	−6.66	0.85
**Validation Set**	***M*_W_^b^**	**ΔΔ*H*_MM_^c^**	**ΔΔ*G*_sol_^d^**	**ΔΔ*TS*_vib_^e^**	**ΔΔ*G*_com_^f^**	**p*IC*_50_^pre^/p*IC*_50_^exp^^h^**
	**(g·mol^−1^)**	**(kcal·mol^−1^)**	**(kcal·mol^−1^)**	**(kcal·mol^−1^)**	**(kcal·mol^−1^)**	
PCAM21	315	0.12	−0.16	0.37	−0.41	1.069
PCAM22	320	4.43	−0.01	−1.90	6.33	1.016
PCAM23	379	2.27	−0.61	−1.94	3.60	1.013

^a^ for the chemical structures of the training set of inhibitors see Table 1; ^b^ *M*_w_ is the molecular mass of inhibitors; ^c^ ΔΔ*H*_MM_ is the relative enthalpic contribution to the Gibbs free energy change related to enzyme-inhibitor (E:I) complex formation derived by molecular mechanics (MM): ΔΔ*H*_MM_ ≅ [*E*_MM_{E:I_x_} − *E*_MM_{I_x_}] − [*E*_MM_{E:I_ref_} − *E*_MM_{I_ref_}], I_ref_ is the reference inhibitor PCAM1; ^d^ ΔΔ*G*_sol_ is the relative solvation Gibbs free energy contribution to the Gibbs free energy change of E:I complex formation: ΔΔ*G*_sol_ = [*G*_sol_{E:I_x_} − *G*_sol_{I_x_}] − [*G*_sol_{E:I_ref_} − *G*_sol_{I_ref_}]; ^e^ ΔΔ*TS*_vib_ is the relative entropic contribution of inhibitor I_x_ to the Gibbs free energy related to E:I complex formation: ΔΔ*TS*_vib_ = [ΔΔ*TS*_vib_{I_x_}_E_ − ΔΔ*TS*_vib_{I_x_}] − [ΔΔ*TS*_vib_{I_ref_}_E_ − ΔΔ*TS*_vib_{I_ref_}]; ^f^ ΔΔ*G*_com_ ≅ ΔΔ*H*_MM_ + ΔΔ*G*_sol_ − ΔΔ*TS*_vib_ is the relative Gibbs free energy change related to E:I_x_ complex formation:; ^g^ *IC*_50_^exp^ is the experimental half-maximal inhibitory concentration of InhA inhibition obtained from reference [12]; ^h^ Ratio of predicted and experimental half-maximal inhibition concentrations p*IC*_50_^pre^/p*IC*_50_^exp^ (p*IC*_50_^pre^ = −log_10_*IC*_50_^pre^) was predicted from computed ΔΔ*G*_com_ using the regression equation for InhA shown in Table 3.

**Figure 4:**
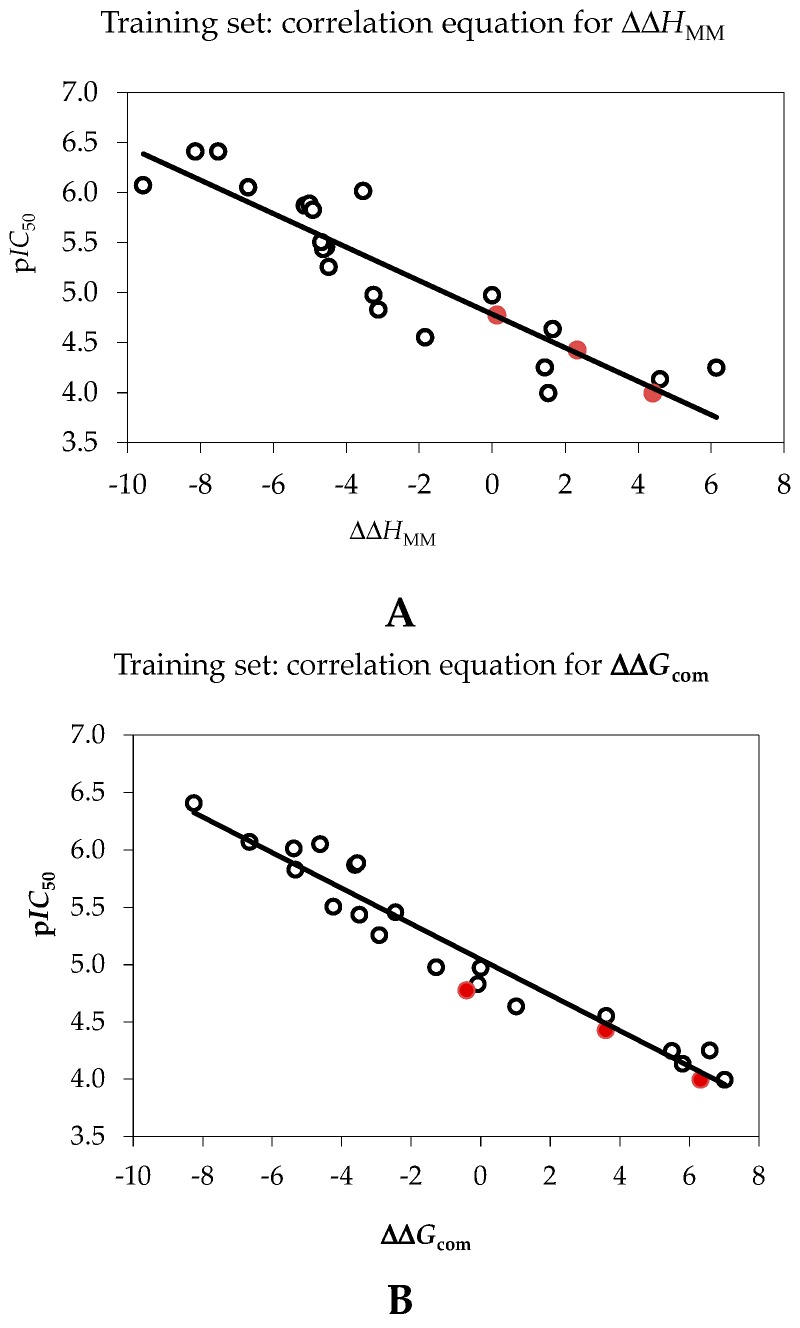
(**A**) Plot of correlation equation between p*IC*_50_ and relative enthalpic contribution to the Gibbs free energy of InhA-PCAMx complex formation (Equation (6)) ΔΔ*H*_MM_; (**B**) Similar plot for relative complexation Gibbs free energies of the InhA-PCAMx complex formation ΔΔ*G*_com_ of the training set, all in kcal·mol^−1^. The data for the validation set are shown in red color; the black circles represent PCAMs from the training set.

Since the ΔΔ*G*_com_ was computed using an approximate approach, the relevance of the binding model was evaluated via correlation with the observed activity data (*IC*_50_^exp^, [12]) by linear regression, Equation (8), Section 3.6. Two correlation equations obtained for the Gibbs free energy of enzyme-inhibitor complex formation ΔΔ*G*_com_ and its enthalpic component ΔΔ*H*_MM_ are shown in Table 3 together with the relevant statistical data. The correlations are plotted on Figure 4. Relatively high values of the regression coefficient and Fischer *F*-*test* of the correlation involving ΔΔ*G*_com_, Equation (B), Table 3, indicate that there is a strong relationship between the binding model and the experimental inhibitory potencies of the series of PCAMs.

**Table 3:** Regression analysis of computed binding affinities ΔΔ*G*_com_, its enthalpic component ΔΔ*H*_MM_, and experimental half-maximal inhibitory concentrations p*IC*_50_^exp^ = −log_10_*IC*_50_^exp^ [12] of pyrrolidine carboxamides (PCAMs) towards InhA of *Mycobacterium tuberculosis* (*MTb*).

Statistical Data of Linear Regression	(A)	(B)
p*IC*_50_^exp^ = −0.1674·ΔΔ*H*_MM_ + 4.7843 (A)	–	–
p*IC*_50_^exp^ = −0.1552·ΔΔ*G*_com_ + 5.0448 (B)	–	–
Number of compounds *n*	20	20
Square correlation coefficient of regression *R*^2^	0.80	0.94
LOO cross-validated square correlation coefficient *R*^2^_xv_	0.76	0.93
Standard error of regression σ	0.38	0.22
Statistical significance of regression, Fisher *F-test*	52.7	199.8
Level of statistical significance α	>95%	>95%
Range of activities *IC*_50_^exp^ (μM)	0.39–101.0	

The ratio of predicted and observed inhibitory potencies (p*IC*_50_^pre^/p*IC*_50_^exp^, the p*IC*_50_^pre^ was calculated using correlation Equation (B), Table 3) for validation set of PCAMs not included into the training set, is close to one and documents considerable predictive power of the complexation QSAR model, Table 2. Therefore, the correlation Equation (B) and computed ΔΔ*G*_com_ quantities can be used for prediction of inhibitory potencies *IC*_50_^pre^ against InhA of *MTb* for novel PCAM analogues, provided that they share the same binding mode as the training set of pyrrolidine carboxamides.

**Figure 5:**
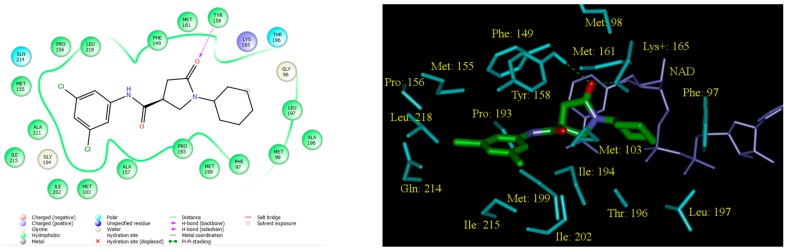
(**Left**) 2D schematic interaction diagram of the most potent inhibitor PCAM9 (Table 1) [12] at the active-site of InhA of *MTb*; (**Right**) 3D structure of the active-site with bound inhibitor PCAM9.

In the crystal structure of InhA-PCAM1 [12] the benzene ring of the inhibitor sits in a hydrophobic cavity of the active-site surrounded by side chains of predominantly nonpolar residues: Met103, Gly104, Phe149, Met155, Pro156, Ala157, Tyr158, Pro193, Met199, Ile202, Leu207, Ala211, Gln214, Ile215 and Leu218, Figure 5 [9,14]. Ring substitutions in the *meta* position by a halogen atom (F, Cl or Br), which form van der Waal contacts to residues Met103, Ala157, Tyr158, Leu202 and Ile215 one side and Phe149, Met155 and Leu218 on the other side of the benzene ring, enhanced observed inhibitory potencies of PCAMs. Double substitution by chlorine atoms in *meta* positions yielded the most active inhibitor PCAM9. Besides halogen atoms also substitutions by smaller nonpolar groups such as methyl, methoxy or isopropyl group in the *meta* position resulted in somewhat elevated potencies compared to PCAM1. On the other hand, substitutions by small to moderate size electron-withdrawing groups in *ortho* and *para* positions decreased the inhibitory potencies of PCAMs substantially, Table 1 [14].

The CoMFA and CoMSIA analyses of PCAM inhibitors proposed useful structural information facilitating the design of new effective antituberculotic agents, namely the aromatic ring that can fit into the larger hydrophobic pocket of InhA should contain judicious substitution(s) by nucleophilic groups [14]. In fact as we can see from Table 1, for the three bromo substituted compounds PCAM8, PCAM3 and PCAM2 a change of the bromine atom from position 2 (PCAM8, *IC*_50_^exp^ = 101 μM) to position 3 (PCAM3, *IC*_50_^exp^ = 0.89 μM) and finally to position 4 (PCAM2, *IC*_50_^exp^ = 28 μM) [12] resulted in a significant variation of the inhibitory potencies. It can be pointed out that the ten most active training set compounds with *IC*_50_^exp^ lower than 4 μM are substituted in positions 3 or 5 or both except PCAM16, which contains a 2-CH_3_, 5-Cl substituents on its benzene ring.

**Figure 6:**
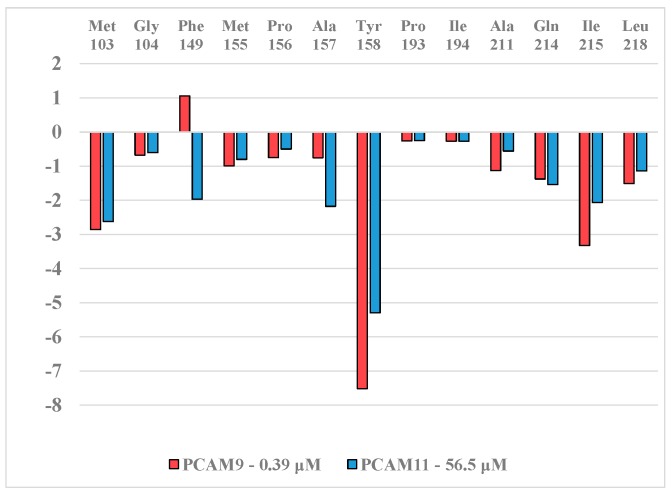
Molecular mechanics intermolecular interaction energy *E*_int_ breakdown (in kcal·mol^−1^) to residue contributions for PCAM9 and PCAM11, Table 2 [12].

In order to identify structural modifications of the benzene ring leading to increased binding affinity of PCAMs to InhA of *MTb* we have carried out detailed analysis of interactions in a series of InhA-PCAMs complexes with help of the complexation QSAR model. The first step of this analysis aimed at obtaining insight into InhA active-site interactions by performing the interaction energy breakdown into contributions from individual residues filling the hydrophobic pocket displayed on Figure 5 for the most active inhibitor and an inactive inhibitor PCAM9 and PCAM11, respectively, Table 2 [12]. The results of the interaction energy analysis are shown on Figure 6.

Figure 6 shows the dependence of *E*_int_ on substitutions of the benzene ring of PCAMs by comparing the potent 3,5-dichloro pyrrolidine carboxamide PCAM9 (*IC*_50_^exp^ = 0.39 μM, Table 2) and 2,5-dichlorophenyl analogue PCAM11 (*IC*_50_^exp^ = 56.5 μM) [12]. Noticeable differences were observed for the active-site residues Ile215 and Tyr158 favoring the interaction with PCAM9 *vs.* Phe149 and Ala157 favoring the PCAM11. The effect of moving one Cl substituent in PCAM9 from *meta* to *ortho* position resulted in a 145-fold decrease of the inhibitory potency. In our recent study on thymine like inhibitors of thymidine monophosphate kinase (TMPK) of *MTb*, we were able to discriminate between active and inactive analogues based on the breakdown of *E*_int_ into individual contributions from active-site residues [16]. The same approach applied to PCAMs in order to access the influence of halogen substitution over the biological activity is not as conclusive as previously about the suggestion of structural modifications able to lead to novel more potent PCAMs.

### 2.2. Ligand-Based 3D-QSAR Pharmacophore Model of Inhibitory Activity

The process of 3D-QSAR PH4 pharmacophore generation was carried out in three steps: the constructive step, the subtractive step, and the optimization step. During the constructive phase of HypoGen the most active compounds for which *IC*_50_^exp^ ≤ 2 × 0.39 μM, were automatically selected as the leads. Thus, the most active PCAM9 (*IC*_50_^exp^ = 0.39 μM) alone was used to generate the starting PH4 features. The features, which matched this lead, were retained. During the subsequent subtractive phase, pharmacophoric features which were present in more than half of the inactive compounds were removed. The pharmacophores which contained all features were retained. None of the training set compounds was found to be inactive (*IC*_50_^exp^ > 0.39 × 10^3.5^ μM = 1233.3 μM). During the final optimization phase, the score of the pharmacophoric hypotheses was improved. Hypotheses are scored via simulated annealing approach according to errors in the activity estimates from regression and complexity. At the end of the optimization, 10 best scoring unique pharmacophoric hypotheses displaying four features were kept.

The reliability of the generated pharmacophore models was then assessed using the calculated cost parameters. The overall costs ranged from 64.6 (Hypo1) to 115.6 (Hypo10). The gap between the highest and lowest cost parameter was relatively small and matched well with the homogeneity of the generated hypotheses and consistency of the training set. For the best PH4 model the fixed cost (40.1) was lower than the null cost (217.4) by Δ = 177.3. This difference represents a chief indicator of the predictability of the PH4 model (Δ > 70 corresponds to a probability higher than 90% that the model represents a valid correlation [16]). In order to be statistically significant the hypotheses have to reach values as similar as possible to the fixed cost and as different as possible from the null cost. The difference Δ ≥ 101.8 for the set of 10 hypotheses confirms high quality of the pharmacophore model. The standard indicator as the root-mean-square deviation (RMSD) between the hypotheses ranged from 1.827 to 3.138 while the squared correlation coefficient (*R*^2^) occupied an interval from 0.63 to 0.88. The PH4 hypothesis with the best RMSD and highest *R*^2^ was retained for further analysis. The statistical data for the set of hypotheses (costs, RMSD, *R*^2^) are listed in Table 4.

**Figure 7:**
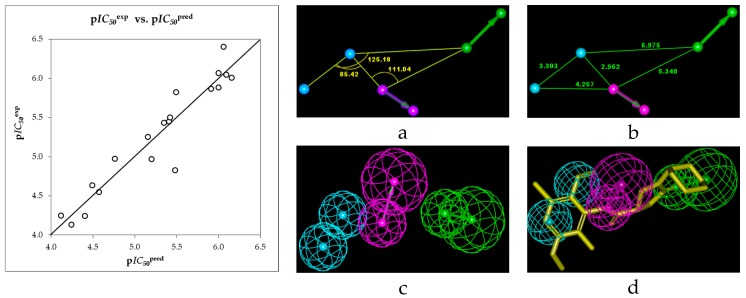
Coordinates (**a**,**b**), features (**c**) and mapping (**d**) of the InhA inhibitor pharmacophore with the best fit hit 21-2-H-6-4 (yellow). The correlation plot of experimental *vs.* predicted inhibitory activity is displayed at the left. The features are colored blue for hydrophobic aliphatic (HYd), green for hydrogen-bond acceptor (HBA) and purple for hydrogen-bond donor (HBD). The arrows represent the projection for donor and acceptor features.

The geometry of the Hypo1 pharmacophore of InhA inhibition is displayed on Figure 7. The regression equation for p*IC*_50_^exp^ *vs.* p*IC*_50_^pre^ estimated from Hypo1: p*IC*_50_^exp^ = 0.9237·p*IC*_50_^pre^ + 0.393 (*n* = 20, *R*^2^ = 0.91, *R*_xv_^2^ = 0.9, *F*-*test* = 191.5, σ = 0.224, α > 95%) is also plotted on Figure 7. To check the predictive power of the generated pharmacophore model we have computed the ratio of predicted and observed activities (p*IC*_50_^pre^/p*IC*_50_^exp^) for the validation set, Table 1. The computed ratios are as follows: PCAM21: 1.261, PCAM22: 1.017, PCAM23: 0.991; all of them displayed values of the ratio relatively close to one, which confirms the substantial predictive power of this regression for the best PH4 model.

**Table 4:** Output parameters of 10 generated PH4 pharmacophoric hypotheses for InhA inhibitors after CatScramble validation procedure.

Hypothesis	*RMSD* ^a^	*R*^2^ ^b^	Total Costs ^c^
Hypo1	1.827	0.88	64.6
Hypo2	1.887	0.87	65.9
Hypo3	2.561	0.75	86.1
Hypo4	2.744	0.72	93.7
Hypo5	2.762	0.71	93.9
Hypo6	2.725	0.72	94.8
Hypo7	3.144	0.63	113.7
Hypo8	3.144	0.63	113.7
Hypo9	3.172	0.62	115.1
Hypo10	3.138	0.63	115.6
Fixed Cost	0.0	1.0	40.1
Null Cost	5.167	0.0	217.4

^a^ root mean square deviation (*RMSD*); ^b^ squared correlation coefficient; ^c^ overall cost parameter of the PH4 pharmacophore.

The validation of the PH4 model was carried out by randomization using the CatScramble algorithm of Catalyst for 49 random runs corresponding to a 98% confidence level. This validation procedure created 10 hypotheses for each run. However, all of them were less predictive then the Hypo10, the hypothesis with the highest cost out of the ten best-generated hypotheses, Table 4. Thus, the best-selected hypothesis Hypo1 represents with a probability of 98% a pharmacophore model of the inhibitory activity of PCAMs with a similar level of predictive power as the complexation QSAR model, which relies on the computed Gibbs free energies of enzyme-inhibitor binding.

Next we have performed computational design and selection of new PCAM analogues with increased inhibition potencies against InhA of *MTb*. The design strategy relied on the hydrophobic feature included in the best PH4 pharmacophore model at the position of the benzene ring coupled with mapping of the ring substitutions to the hydrophobic features in the PH4 hypothesis Hypo1 (Figure 7).

### 2.3. Library Design and Adsorption, Distribution, Metabolism and Excretion (ADME) Focusing

We have built a virtual library of new pyrrolidine carboxamide compounds with a variety of substitutions in five positions of the benzene ring with the goal to identify more potent orally bioavailable inhibitors of the InhA of *MTb*. PCAM analogues with substituted phenyl ring can be prepared through amide synthesis from pyrrolidine carboxylic acid and substituted anilines following a previously published protocol [12]. During the virtual library enumeration the R-groups listed in Table 5 were attached to positions R_1_–R_5_ of the benzene ring of the PCAM scaffold to form an combinatorial library of the size: R_1_ × R_2_ × R_3_ × R_4_ × R_5_ = 31 × 26 × 27 × 26 × 31 = 17,540,172 analogues. In order to match the substitution pattern of the best training set inhibitor PCAM9 and to take into account the reported structural information about *para* position that is not suitable for substitutions [12]. Hydrogen atom was added to the list of R_3_-group bringing it to 27 fragments. This subset of 649,636 virtual PCAMs is expected to yield the best new PCAM analogues with activity better than the 0.39 μM potency of PCAM9. In order to avoid steric effects, bulkier substituents were used only at extreme positions R_1_ and R_5_. Including them at all positions is detrimental since they will be excluded through the Lipinski’s rule violation (molecular weight >500 g·mol^−1^) [17]. Relatively large initial diversity library of PCAM analogues was generated from the substituted anilines listed in the databases of available chemicals [18]. In order to design a more focused library of a reduced size and increased content of drug-like and orally bioavailable molecules, we have introduced a set of filters and penalties, which can help to select smaller number of suitable PCAMs that can be submitted to *in silico* screening. The initial virtual library was thus filtered in an ADME-based focusing step to remove compounds with expected poor oral bioavailability and low drug-like character. Only analogues with high predicted percentage of human oral absorption (HOA) in the gastrointestinal tract larger than 80% [19,20] and compounds satisfying the Lipinski’s rule of five [17] computed for the entire virtual library using QikProp software [21], were kept. This focusing has reduced the size of the initial library to 1,604,448 analogues, less than 10% of its original number size.

**Table 5:** R-groups (fragments, building blocks, substituents) used in the design of the initial diversity library of PCAM analogues.

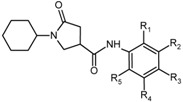					
R-Groups ^a,b^					
1	–F	2.	–Cl	3.	–Br
4.	–CH_3_	5.	–C_2_H_5_	6.	–OCH_3_
7.	–OC_2_H_5_	8.	–CH_2_F	9.	–CH_2_Cl
10.	–CH_2_Br	11.	–COOMe	12.	–CHO
13.	–OH	14.	–NO_2_	15.	–NO
16.	–CN	17.	–SO_2_H	18.	–CF_3_
19.	–NCS	20.	–C≡CH	21.	–CH=CH_2_
22.	–NH_2_	23.	–CONH_2_	24.	–C(CH_3_)=CH_2_
25.	–I	26.	*i*-Prop	27.	–CH_2_I
28.	–SH	29.	–CH=C=CH_2_	30.	–CH_2_–CHO
31.	–SO_2_NH_2_				

^a^ fragments 1–26 were used in R_2_ and R_4_-groups; fragments 1–26 and H were used in R_3_-group and fragments 1–31 were used in R_1_ and R_5_-groups; ^b^ dashed bonds indicates the attachment points of individual fragments.

The selected library subset then underwent virtual screening by means of the PH4 pharmacophore of PCAM inhibitory activity towards model of InhA of *MTb*.

### 2.4. In Silico Screening of Library of PCAMs

The library of PCAM analogues was further screened for molecular structures matching to the 3D-QSAR PH4 pharmacophore model Hypo1 of InhA inhibition. From the set of 1,604,448 analogues few thousands of PCAMs mapped to at least 2 features, 592 of which mapped to 4 features of the pharmacophore. Out of then, only 115 best fitting analogues (PH4 hits) have been retained and submitted to screening with help of the complexation QSAR model. Computed Gibbs free energy of complex formation with InhA of *MTb* and its component as well as predicted half-maximal inhibitory concentrations *IC*_50_^pre^ estimated from the correlation Equation (B), Table 3, are given in Table 6.

For the majority of new PCAM analogues, the estimated inhibitory potencies shown in Table 6 are better than that for the most active training set compound PCAM9 (*IC*_50_^exp^ = 390 nM [12]). In fact, for the best-designed PCAM analogue 21-2-H-6-4 the predicted inhibitory potency is more than 80 times higher than for the PCAM9.

### 2.5. Analysis of New Inhibitors

**Figure 8:**
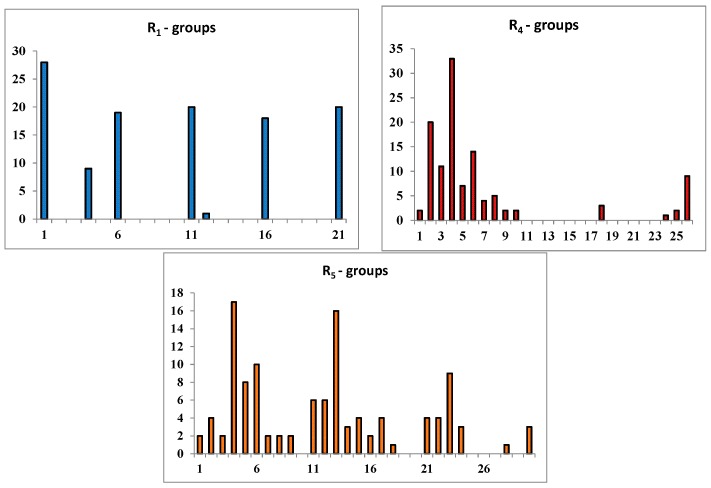
Histograms of frequency of occurrence of individual R_1_, R_4_, R_5_-groups in the 115 best-selected analogues mapping to the four features of the PH4 pharmacophore hypothesis Hypo1 (for fragments’ structures see Table 5); For R_2_ and R_3_ the occurrences are indicated in parenthesis: R_2_ = –F (14), –Cl (97), –C≡CH (3), –NH_2_ (1) and R_3_ = –F (46), –H (69).

In order to identify which substituents on the benzene ring of PCAMs (Table 5) lead to new inhibitor candidates with the highest predicted potencies towards the InhA of *MTb*, we have prepared histograms of the frequency of occurrence of R_1_, R_4_ and R_5_ groups in the 115 PH4 best fitting hit PCAMs selected from the focused virtual library shown in Table 6 (Figure 8).

**Table 6:** Complexation Gibbs free energies and their components for the top 100 scoring virtually designed PCAM analogues. The analogue numbering concatenates the index of each substituent R_1_ to R_5_ with the substituent numbers taken from Table 5 except for hydrogen which is directly specified by the letter H.

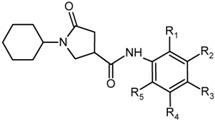											
Designed Analogues	Substituents					*M*_W_^a^	ΔΔ*H*_MM_^b^	ΔΔ*G*_sol_^c^	ΔΔ*TS*_vib_^d^	ΔΔ*G*_com_^e^	*IC*_50_^pre^ ^f^
	R_1_	R_2_	R_3_	R_4_	R_5_	(g/mol)	(kcal/mol)	(kcal/mol)	(kcal/mol)	(kcal/mol)	(nM)
PCAM1	–H	–H	–H	–H	–H	286	0	0	0	0	10,660.0 ^g^
1-2-1-4-2	–F	–Cl	–F	–CH_3_	–Cl	405	−7.15	−8.21	−4.81	−10.54	206.9
1-2-1-4-5	–F	–Cl	–F	–CH_3_	–C_2_H_5_	399	−6.89	−8.12	−0.84	−14.17	56.4
1-2-1-4-21	–F	–Cl	–F	–CH_3_	–CH=CH_2_	397	−8.56	−8.34	−1.98	−14.92	43.1
1-2-1-6-13	–F	–Cl	–F	–OCH_3_	–OH	403	−11.63	−6.95	−2.97	−15.62	33.6
1-2-1-7-14	–F	–Cl	–F	–OC_2_H_5_	–NO_2_	446	−13.30	−5.87	−4.26	−14.91	43.2
1-2-1-26-13	–F	–Cl	–F	*i-*Propyl	–OH	415	−7.39	−7.23	−1.95	−12.67	96.4
1-2-1-26-23	–F	–Cl	–F	*i-*Propyl	–CONH_2_	442	−11.98	−5.41	−2.76	−14.63	47.8
6-2-1-2-5	–OCH_3_	–Cl	–F	–Cl	–C_2_H_5_	431	−11.44	−6.06	−2.61	−14.89	43.5
6-2-1-2-13	–OCH_3_	–Cl	–F	–Cl	–OH	419	−7.90	−7.19	−3.28	−11.81	131.3
6-2-1-2-23	–OCH_3_	–Cl	–F	–Cl	–CONH_2_	446	−9.78	−6.24	−3.20	−12.82	91.4
6-2-1-3-13	–OCH_3_	–Cl	–F	–Br	–OH	464	−9.32	−7.29	−3.07	−13.53	70.77
6-2-1-3-23	–OCH_3_	–Cl	–F	–Br	–CONH_2_	491	−10.96	−6.86	−3.81	−14.01	59.6
6-2-1-4-4	–OCH_3_	–Cl	–F	–CH_3_	–CH_3_	397	−8.95	−6.58	−1.27	−14.26	54.5
6-2-1-4-7	–OCH_3_	–Cl	–F	–CH_3_	–OC_2_H_5_	427	−12.34	−7.83	−1.29	−18.88	10.5
6-2-1-4-9	–OCH_3_	–Cl	–F	–CH_3_	–CH_2_Cl	431	−8.45	−7.98	−2.35	−14.08	58.2
11-2-1-5-3	–COOCH_3_	–Cl	–F	–C_2_H_5_	–Br	506	−11.72	−8.11	−3.69	−16.15	27.8
11-2-1-5-4	–COOCH_3_	–Cl	–F	–C_2_H_5_	–CH_3_	439	−11.24	−8.48	−0.5	−19.22	8.0
11-2-1-6-24	–COOCH_3_	–Cl	–F	–OCH_3_	–CH_3_C=CH_2_	467	−5.86	−7.59	−3.01	−10.45	213.8
11-2-1-8-6	–COOCH_3_	–Cl	–F	–CH_2_F	–OCH_3_	459	−9.65	−5.65	−2.94	−12.36	107.6
11-2-1-9-5	–COOCH_3_	–Cl	–F	–CH_2_Cl	–C_2_H_5_	473	−11.07	−6.91	−2.91	−15.07	40.8
11-2-1-10-1	–COOCH_3_	–Cl	–F	––CH_2_Br	–F	459	−12.63	−8.52	−4.26	−16.89	21.3
11-2-1-26-4	–COOCH_3_	–Cl	–F	*i-*Propyl	–CH_3_	453	−16.25	−3.61	−1.76	−18.10	13.8
16-2-1-2-4	–CN	–Cl	–F	–Cl	–CH_3_	412	−6.60	−7.88	−3.67	−10.82	187.3
16-2-1-4-6	–CN	–Cl	–F	–CH_3_	–OCH_3_	408	−8.94	−6.86	−1.48	−14.33	53.3
16-2-1-4-8	–CN	–Cl	–F	–CH_3_	–CH_2_F	410	−6.51	−6.86	−1.25	−12.12	117.3
16-2-1-4-12	–CN	–Cl	–F	–CH_3_	–CH_2_O	406	−7.99	−7.27	−0.42	−14.84	44.3
21-2-1-2-22	–CH=CH_2_	–Cl	–F	–Cl	–NH_2_	414	−7.52	−7.66	−1.86	−13.32	76.3
21-2-1-4-4	–CH=CH_2_	–Cl	–F	–CH_3_	–CH_3_	393	−7.90	−7.60	0.29	−15.79	31.5
21-2-1-4-12	–CH=CH_2_	–Cl	–F	–CH_3_	–CH_2_O	407	−7.39	−6.49	0.01	−13.89	62.3
21-2-1-4-23	–CH=CH_2_	–Cl	–F	–CH_3_	–CONH_2_	422	−8.40	−5.33	−2.25	−11.48	147.5
21-2-1-5-13	–CH=CH_2_	–Cl	–F	Et	–OH	409	−8.39	−6.95	0.70	−16.03	28.9
21-2-1-6-2	–CH=CH_2_	–Cl	–F	–OCH_3_	–Cl	429	−11.45	−8.04	−2.59	−16.89	21.3
21-2-1-6-4	–CH=CH_2_	–Cl	–F	–OCH_3_	–CH_3_	409	−10.85	−8.45	0.47	−19.77	7.6
21-2-1-6-11	–CH=CH_2_	–Cl	–F	–OCH_3_	–COOCH_3_	507	−7.16	−6.30	−1.43	−12.03	121.1
21-2-1-6-17	–CH=CH_2_	–Cl	–F	–OCH_3_	–SO_2_H	459	−14.65	−2.64	−3.36	−13.93	61.5
1-1-H-1-14	–F	–F	–H	–F	–NO_2_	385	−18.52	−2.39	−6.47	−14,44	51.2
1-1-H-1-15	–F	–F	–H	–F	–NO	369	−7.69	−7.78	−5.89	−9.57	292.1
1-1-H-2-15	–F	–F	–H	–Cl	–NO	385	−9.16	−7.72	−6.05	−10.82	186.9
1-1-H-2-21	–F	–F	–H	–Cl	–C=CH_2_	383	−7.02	−7.98	−4.49	−10.51	209.1
1-1-H-2-24	–F	–F	–H	–Cl	–C(CH_3_)=CH_2_	397	−6.92	−7.89	−4.69	−10.12	240.4
1-1-H-4-5	–F	–F	–H	–CH3	–C_2_H_5_	352	−6.40	−7.80	0.57	−14.77	45.5
1-1-H-4-13	–F	–F	–H	–CH3	–OH	362	−5.32	−8.15	−0.74	−12.74	94.1
1-1-H-4-21	–F	–F	–H	–CH_3_	–C=CH_2_	351	−7.80	−8.24	−1.53	−14.52	49.8
1-1-H-4-22	–F	–F	–H	–CH_3_	–NH_2_	368	−5.62	−8.31	−2.13	−11.81	131.4
1-1-H-4-28	–F	–F	–H	–CH_3_	–SH	364	−4.13	−8.38	−2.42	−10.09	242.7
1-2-H-4-5	–F	–Cl	–H	–CH_3_	–C_2_H_5_	381	−6.28	−8.19	−0.17	−14.30	53.8
1-2-H-4-2	–F	–Cl	–H	–CH_3_	–Cl	387	−5.01	−8.47	−3.38	−10.10	241.9
1-2-H-6-13	–F	–Cl	–H	–OCH_3_	–OH	385	−10.90	−7.36	−1.43	−16.83	21.8
1-2-H-4-21	–F	–Cl	–H	–CH_3_	–CH=CH_2_	379	−7.74	−8.20	−0.45	−15.5	35.2
1-2-H-7-11	–F	–Cl	–H	–OC_2_H_5_	–COOCH_3_	419	−7.21	−6.77	−3.33	−10.65	198.5
1-2-H-7-14	–F	–Cl	–H	–OC_2_H_5_	–NO_2_	428	−12.75	−5.81	−2.91	−15.66	33.1
1-2-H-26-13	–F	–Cl	–H	*i-*Propyl	–OH	397	−6.70	−7.30	−0.38	−13.62	68.7
1-2-H-26-17	–F	–Cl	–H	*i-*Propyl	–SO_2_H	445	−9.15	−3.42	−2.01	−10.56	205.2
1-2-H-26-23	–F	–Cl	–H	*i-*Propyl	–CONH_2_	424	−10.85	−5.73	−2.05	−14.53	49.6
4-1-H-24-30	–CH_3_	–F	–H	–C(CH_3_)=CH_2_	–CH_2_–COH	429	−7.43	−6.72	−0.14	−14.01	59.6
4-1-H-25-4	–CH_3_	–F	–H	–I	–CH_3_	400	−4.89	−7.36	1.93	−14.17	56.3
4-1-H-25-30	–CH_3_	–F	–H	–I	–CH_2_–COH	486	−8.42	−7.78	−0.41	−15.79	31.6
4-1-H-26-12	–CH_3_	–F	–H	*i*-Propyl	–CHO	458	−2.75	−9.06	1.77	−13.58	69.6
4-2-H-2-13	–CH_3_	–Cl	–H	–Cl	–OH	388	−4.56	−7.41	−0.60	−11.37	153.7
4-2-H-3-12	–CH_3_	–Cl	–H	–Br	–CHO	385	−7.28	−8.12	−3.03	−12.38	107.2
4-2-H-5-13	–CH_3_	–Cl	–H	–C_2_H_5_	–OH	441	−5.45	−6.93	2.30	−14.68	46.9
4-2-H-6-5	–CH_3_	–Cl	–H	–OCH_3_	–C_2_H_5_	378	−9.68	−8.67	1.54	−19.89	7.3
4-2-H-6-13	–CH_3_	–Cl	–H	–OCH_3_	–OH	381	−8.19	−7.20	0.45	−15.85	30.9
6-2-H-2-5	–OCH_3_	–Cl	–H	–Cl	–C_2_H_5_	413	−7.85	−0.86	−7.64	−14.63	47.8
6-2-H-2-13	–OCH_3_	–Cl	–H	–Cl	–OH	446	−6.72	−7.65	−2.09	−12.27	111.3
6-2-H-2-23	–OCH_3_	–Cl	–H	–Cl	–CONH_2_	428	−7.26	−6	−2.54	−10.72	193.8
6-2-H-3-13	–OCH_3_	–Cl	–H	–Br	–OH	446	−7.31	−7.01	−1.89	−12.43	105.0
6-2-H-3-23	–OCH_3_	–Cl	–H	–Br	–CONH_2_	473	−9.98	−7.14	−2.87	−14.26	54.6
6-2-H-4-4	–OCH_3_	–Cl	–H	–CH_3_	–CH_3_	379	−7.20	−8.37	0.12	−15.69	32.7
6-2-H-4-7	–OCH_3_	–Cl	–H	–CH_3_	–OC_2_H_5_	409	−11.08	−8.04	0.61	−19.70	7.7
6-2-H-4-9	–OCH_3_	–Cl	–H	–CH_3_	–CH_2_Cl	413	−9.52	−1.42	−8.28	−16.39	25.5
6-20-H-18-22	–OCH_3_	–C≡CH	–H	–CF_3_	–NH_2_	393	−10,23	−6,53	−3,70	−13,06	83.9
6-20-H-18-23	–OCH_3_	–C≡CH	–H	–CF_3_	–CONH_2_	423	−12,25	−7,82	−6,80	−13,27	77.9
6-20-H-18-30	–OCH_3_	–C≡CH	–H	–CF_3_	–CH_2_–COH	451	−13,99	−6,50	−3,99	−16,50	24.5
11-2-H-3-6	–COOCH_3_	–Cl	–H	–Br	–OCH_3_	486	−3.82	−7.77	−1.85	−9.74	275.3
11-2-H-5-3	–COOCH_3_	–Cl	–H	–C_2_H_5_	–Br	488	−9.93	−6.99	−1.72	−15.20	39.0
11-2-H-5-4	–COOCH_3_	–Cl	–H	–C_2_H_5_	–CH_3_	421	−11.29	−7.18	0.96	−19.44	8.6
11-2-H-6-24	–COOCH_3_	–Cl	–H	–OCH_3_	–CH_3_C=CH_2_	449	−4.39	−7.49	−1.21	−10.67	197.2
11-2-H-8-4	–COOCH_3_	–Cl	–H	–CH_2_F	–CH_3_	425	−3.13	−6.65	−0.78	−8.99	359.5
11-2-H-8-6	–COOCH_3_	–Cl	–H	–CH_2_F	–OCH_3_	440	−8.96	−5.53	−1.62	−12.97	86.6
11-2-H-9-5	–COOCH_3_	–Cl	–H	–CH_2_Cl	–C_2_H_5_	455	−12.37	−6.85	−2.41	−16.81	21.9
11-2-H-10-1	–COOCH_3_	–Cl	–H	–CH_2_Br	–F	441	−10.44	−9.07	−3.99	−15.52	34.7
11-2-H-26-4	–COOCH_3_	–Cl	–H	*i-*Propyl	–CH_3_	435	−16.30	−1.95	−0.86	−17.38	17.9
12-22-H-8-18	–CHO	–NH_2_	–H	–CH_2_F	–CF_3_	450	−11.56	−7.71	−3.04	−16.23	27
16-2-H-2-4	–CN	–Cl	–H	–Cl	–CH_3_	394	−4.70	−8.52	−2.75	−10.47	211.7
16-2-H-2-6	–CN	–Cl	–H	–Cl	–OCH_3_	410	−6.87	−6.91	−4.40	−9.38	313.2
16-2-H-2-13	–CN	–Cl	–H	–Cl	–OH	396	−5.52	−7.32	−3.61	−9.23	330.8
16-2-H-3-6	–CN	–Cl	–H	–Br	–OCH_3_	455	−8.58	−7.38	−5.32	−10.64	199.3
16-2-H-4-6	––CN	–Cl	–H	–CH_3_	–OCH_3_	390	−7.58	−7.53	−0.1	−15.01	41.7
16-2-H-4-8	–CN	–Cl	–H	–CH_3_	–CH_2_F	392	−7.65	−7.95	−0.77	−14.82	44.6
16-2-H-4-12	–CN	–Cl	–H	–CH_3_	–CH_2_O	388	−5.95	−6.83	0.16	−12.94	87.5
21-2-H-2-22	–CH=CH_2_	–Cl	–H	–Cl	–NH_2_	396	−6.35	−0.83	−7.53	−13.05	84.0
21-2-H-4-4	–CH=CH_2_	–Cl	–H	–CH_3_	–CH_3_	375	−7.31	−7.56	0.97	−15.85	30.9
21-2-H-4-12	–CH=CH_2_	–Cl	–H	–CH_3_	–CH_2_O	389	−8.30	−6.77	0.17	−15.24	38.4
21-2-H-4-23	–CH=CH_2_	–Cl	–H	–CH_3_	–CONH_2_	404	−6.44	−6.28	−0.86	−11.86	128.9
21-2-H-5-13	–CH=CH_2_	–Cl	–H	–C_2_H_5_	–OH	391	−7.09	−7.1	1.34	−15.5	34.6
21-2-H-6-2	–CH=CH_2_	–Cl	–H	–OCH_3_	–Cl	411	−11.55	−8.14	−1.16	−18.5	11.9
21-2-H-6-4	–CH=CH_2_	–Cl	–H	–OCH_3_	–CH_3_	391	−10.70	−8.46	2.05	−21.2	4.5
21-2-H-6-11	–CH=CH_2_	–Cl	–H	–OCH_3_	–COOCH_3_	490	−6.84	−6.24	−0.86	−12.22	113.5
21-2-H-6-17	–CH=CH_2_	–Cl	–H	–OCH_3_	–SO_2_H	441	−12.45	−2.69	−1.52	−13.62	68.6

^a^ *M*_W_ is molecular mass of the inhibitor; ^b^ ΔΔ*H*_MM_ is the relative enthalpic contribution to the Gibbs free energy change related to the InhA**-**PCAM complex formation ΔΔ*G*_com_ (for details see footnote of Table 2); ^c^ ΔΔ*G*_sol_ is the relative solvation Gibbs free energy contribution to ΔΔ*G*_com_; ^d^ ΔΔ*TS*_vib_ is the relative entropic (vibrational) contribution to ΔΔ*G_c_*_om_; ^e^ ΔΔ*G*_com_ is the relative Gibbs free energy change related to the enzyme-inhibitor InhA**-**PCAM complex formation ΔΔ*G*_com_ ≅ ΔΔ*H*_MM_ + ΔΔ*G*_sol_ − ΔΔ*TS*_vib_; ^f^ *IC*_50_^pre^ is the predicted half-maximal inhibitory concentration of PCAMx towards InhA of *MTb* calculated from ΔΔ*G*_com_ using correlation Equation (B), Table 3; ^g^ *IC*_50_^exp^ is given for the reference inhibitor PCAM1 instead of *IC*_50_^pre^.

The R_2_ and R_3_ groups of this highly focused combinatorial subset of the virtual library of PCAMs were: R_2_ = –F (14), –Cl (97), –C≡CH (3), –NH_2_ (1) and R_3_ = –F (46), –H (69). It follows from the histograms in Figure 8 that R_1_ groups –F, –OCH_3_, –COOCH_3_, –CN, and –CH=CH_2_ were represented with almost equal frequencies of occurrence in the PCAMs subset. The R_4_ groups contain preferentially –Cl, –CH_3_, and –OCH_3_ fragments, while the R_5_ groups include chiefly –CH_3_, –OCH_3_, –OH, and –CONH_2_ ones. The top scoring virtual hits are PCAM analogues: 21-2-1-6-4 (7.6 nM), 11-2-1-5-4 (8.0 nM), 6-2-1-4-7 (10.5 nM), 11-2-1-26-4 (13.8 nM), and 21-2-1-6-2 (21.3 nM) for fully substituted benzene ring while 21-2-H-6-4 (4.5 nM), 6-2-H-4-7 (7.7 nM), 11-2-H-5-4 (8.6 nM), 21-2-H-5-13 (11.9 nM), and 4-2-H-6-5 (7.3 nM) for *para* non substituted benzene ring. Best substitutions in the *ortho* positions of the benzene ring (R_1_- or R_5_- groups) include: –OCH_3_ (6), –COOCH_3_ (11), and –CH=CH_2_ (21) (R_1_) or –CH_3_ (4), –OC_2_H_5_ (7), and –OH (13) (R_5_) fragments, respectively. Due to diverse amino acid composition of the hydrophobic pocket, which accommodates the substituted benzene ring, the R_1_ and R_5_ groups display dissimilar preferences for favorable building blocks. Substitutions in the *meta* positions (R_2_- or R_4_-groups) include –Cl atom, which displayed significant contribution to the inhibitory potencies of the training set PCAMs [12] (R_2_) or predominantly hydrophobic fragments –CH_3_ (4), –C_2_H_5_ (5), –OCH_3_ (6), and isopropyl (26) (R_5_). Substitutions in the *para* position (R_3_-group) include –F and –H atoms; this position seems to favor the hydrogen atom [12].

**Figure 9:**
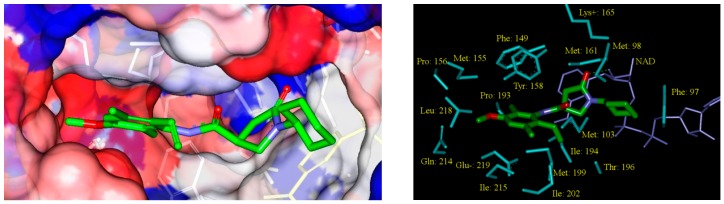
(**Left**) Connolly surface of the active-site of InhA of *MTb* with bound predicted most active PCM inhibitor 21-2-H-6-4. The binding site surface is colored according to residue hydrophobicity: red—hydrophobic, blue—hydrophilic, and white—intermediate; (**Right**) Close up of virtual hit 21-2-H-6-4 at the active-site of InhA. Interacting residues are colored blue and NADH is shown in purple color.

The effect of the substitutions on the benzene ring of PCAMs resulted in an overall increase of inhibitory potencies of the new designed analogues. The predicted activity of the best designed compound 21-2-H-6-4 reached a half-maximal inhibitory concentration approximately 86-times lower than that of the most active compound of the training set PCAM9 (H-2-H-2-H) with *IC*_50_^exp^ = 390 nM, Figure 9.

### 2.6. ADME Profiles of Designed PCAMs

The ADME-related properties described in Section 3.9 were computed. The values for the best active designed PCAMs were compared with those computed for drugs used for treatment of tuberculosis or currently undergoing clinical trials, Table 7. The best designed analogues all display #stars descriptor equal to zero, meaning that the optimal value range of none of the drug-likeness descriptors was violated. Thus, the designed PCAMs are predicted to display high level of drug- likeness as well as human oral absorption in the gastrointestinal tract (HOA).

**Table 7:** Predicted ADME-related properties of the best-designed PCAM analogues and known antituberculotic agents either in clinical use or currently undergoing clinical testing, as computed by QikProp [21].

PCAM ^a^	#stars ^b^	*M*_W_^c^ (g·mol^−1^)	*S*_mol_^d^ (Å^2^)	*S*_mol, hfo_^e^ (Å^2^)	*V*_mol_^f^ (Å^3^)	*Rot*B ^g^	*HB*_don_^h^	*HB*_acc_^i^	log*P*_o/w_^j^	log*S*_wat_^k^	log*K*_HSA_^l^	log*B/B* ^m^	BIP_caco_^n^ (nm·s^−1^)	*#meta* °	*IC*_50_^pre^ ^p^	HOA ^q^	%HOA ^r^
1-2-1-6-13	0	402.8	663.6	395.3	1169	4	2	7	2.7	−4.4	−0.08	−0.52	452.2	4	33.6	3	90.1
6-2-1-4-7	0	426.9	685	509.6	1285	5	1	7	3.8	−4.5	0.20	−0.06	1434.6	5	10.5	3	100
21-2-1-4-4	0	392.9	676	481.4	1237	3	1	5.5	4.1	−5.2	0.46	0.04	1414.3	4	31.5	3	100
11-2-1-5-4	0	438.9	702.2	504.3	1313.1	4	0	6.5	3.8	−4.6	0.2	−0.3	721.4	4	8.0	3	100
21-2-1-6-2	0	429.3	686	441.9	1251	4	1	6.3	4.1	−5.2	0.30	0.09	1414.3	3	21.3	3	100
21-2-1-5-13	0	408.9	695.5	472.8	1265	5	2	6.3	3.6	−4.9	0.27	−0.49	628.0	4	28.9	3	100
21-2-1-6-4	0	408.9	684.3	500.5	1260	4	1	6.3	3.9	−4.9	0.32	−0.04	1426.4	4	7.6	3	100
11-2-1-26-4	0	453	706.9	528.1	1346	4	0	6.5	4.0	−4.7	0.30	-0.28	840.5	4	13.8	3	100
21-2-H-6-4	0	390.9	698.2	521.8	1261	4	1	6.3	3.9	−5	0.32	−0.10	1467.3	5	4.5	3	100
6-2-H-4-7	0	408.9	689.8	524.4	1286	5	1	7	3.6	−4.4	0.20	−0.19	1311.2	6	7.7	3	100
21-2-H-6-2	0	411.3	688.7	445.5	1244	4	1	6.3	4.0	−5.1	0.27	0.04	1413.9	4	11.9	3	100
21-2-H-5-13	0	390.9	691.5	476.4	1255	5	2	6.3	3.4	−4.7	0.24	−0.54	631.1	5	34.6	3	100
21-2-H-4-4	0	374.9	691.9	506.2	1249	3	1	5.5	4.1	−5.4	0.48	−0.04	1441.3	5	30.9	3	100
1-2-H-4-21	0	378.9	660.2	428.2	1194	3	1	5.5	3.9	−5	0.34	0.01	1321.1	4	35.2	3	100
6-2-H-2-5	0	413.3	671.4	440.2	1241	4	1	6.3	4.0	−4.8	0.28	0.05	1389.1	5	47.8	3	100
1-2-H-6-13	0	384.8	656.6	397.5	1155	4	2	7	2.5	−4.2	−0.11	−0.56	452.7	5	21.8	3	89.3
1-2-H-7-14	0	427.9	721.2	435.4	1277	5	1	7.3	3.0	−5	0.10	−1.05	214.6	5	33.1	3	86.4
11-2-H-5-4	0	420.9	698.4	509.7	1304	4	0	6.5	3.7	−4.4	0.17	−0.38	748.6	5	8.6	3	100
11-2-H-26-4	0	435	707.2	534.8	1340	4	0	6.5	3.9	−4.6	0.28	−0.33	825.5	5	17.9	3	100
11-2-H-9-5	0	455	697.2	448.7	1326	4	0	6.5	4.1	−4.7	0.24	−0.18	780.0	5	21.9	3	100
4-1-H-25-30	0	486.3	672.7	386.1	1209	4	1	7.5	2.7	−4.7	−0.07	−0.661	410.6	5	31.6	3	89.6
4-2-H-6-5	0	392.9	686.7	527.2	1241	4	1	6.2	3.7	−5.2	0.27	−0.126	1675.4	6	7.3	3	100
4-2-H-6-13	0	380.8	662.2	451.1	1173	4	2	7	2.5	−4.2	−0.06	−0.576	532.9	6	30.9	3	90.7
6-20-H-18-30	0	450.4	727.9	446.8	1329	6	1.5	8.2	3.2	−5.1	0.02	−0.632	625.9	5	24.5	3	95.9
12-22-H-8-18	0	429.4	662.7	341.8	1201	4	1	6.5	2.9	−4.9	0.12	−0.885	216.7	5	27	3	85.9
11-2-H-10-1	0	489.8	685.6	380.4	1265	4	0	6.5	3.8	−4.6	0.06	−0.157	700.7	4	34.7	3	100
11-2-H-5-3	0	485.8	702.6	457.6	1304	4	0	6.5	3.8	−4.7	0.17	−0.296	703.9	4	39	3	100
Rifampin	1	137.1	313.3	0.0	479.2 *	2	3	4.5	−0.7	0	−0.8	−0.8	267.5	2	-	2	67
Isoniazid	4	123.1 *	299.3	0.0	442.6 *	1	2	5	−0.6	−0.5	−0.8	−0.7	298.4	4	-	2	67
Ethambutol	2	204.3	475.7	395.8	805.7	11	4	6.4	−0.2	0.6	−0.8	0.0	107.8	4	-	2	62
Pyrazinamide	10	823.0 *	1090 *	850.0 *	2300 *	25 *	6	20.3 *	3.0	−3.1	−0.3	−2.7	38.2	11 *	-	1	34
Gatifloxacin	0	375.4	597.5	355.7	1093.0	2	1	6.8	0.5	−4.0	0	−0.6	17.0	1	-	2	52
Moxifloxacin	0	401.4	641.2	395.6	1167.1	2	1	6.8	1.0	−4.7	0.2	−0.6	20.9	1	-	2	56
Rifapentine	10	877.0 *	1024.3 *	844.9 *	2332.6 *	24 *	6	20.9 *	3.6	−2.2	−0.2	−1.5	224.0	13 *	-	1	51
Bedaquiline	4	555.5	786.5	213.7	1531.7	9	1	3.8	7.6^*^	−6.9	1.7	0.4	1562.2	5	-	1	100
Delamanid	2	534.5	795.6	284.4	1469.9	7	0	6.0	5.8	−7.6	1.0	−1.0	590.9	2	-	1	85
Linezolid	0	337.4	554.6	337.2	995.4	2	1	8.7	0.6	−2.0	−0.7	−0.5	507.0	2	-	3	79
Sutezolid	1	353.4	594.0	330.6	1046.2	2	1	7.5	1.3	−3.4	−0.4	−0.4	449.3	0	-	3	82
Ofloxacin	1	361.4	580.5	337.0	1044.0	1	0	7.3	−0.4	−2.8	−0.5	−0.4	25.9	1	-	2	50
Amikacin	14	585.6	738.3	350.3	1499.5	22 *	17 *	26.9 *	−7.9 *	−0.2	−2.1	−3.5	0	14 *	-	1	0
Kanamycin	10	484.5	655.8	258.9	1290.9	17 *	15 *	22.7 *	−6.7 *	2.0	−1.4	−3.1	0	12 *	-	1	0
Imipenem	0	299.3	486.5	259.1	879.4	8	3	7.2	1.0	−1.8	−0.7	−1.4	35.0	3	-	3	61
Amoxicillin	2	365.4	560.8	164.6	1032.9	6	4.25	8.0	−2.5	−0.8	−1.1	−1.5	1.0	5	-	1	12
Clavulanate	0	199.2	396.1	184.6	629.5	4	2	6.5	−0.8	0.3	−1.3	−1.3	13.3	2	-	2	42

^a^ designed PCAM analogues, Table 6; ^b^ drug-likeness, number of property descriptors (from 24 out of the full list of 49 descriptors of QikProp, ver. 3.7, release 14) that fall outside of the range of values for 95% of known drugs; ^c^ molecular weight in g·mol^−1^ (range for 95% of drugs: 130–725 g·mol^−1^) [21]; ^d^ total solvent-accessible molecular surface, in Å^2^ (probe radius 1.4 Å) (range for 95% of drugs: 300–1000 Å^2^); ^e^ hydrophobic (hfo) portion of the solvent-accessible molecular (mol) surface, in Å^2^ (probe radius 1.4 Å) (range for 95% of drugs: 0–750 Å^2^); ^f^ total volume of molecule (mol) enclosed by solvent-accessible molecular surface, in Å^3^ (probe radius 1.4 Å) (range for 95% of drugs: 500–2000 Å^3^); ^g^ number of non-trivial (not CX3), non-hindered (not alkene, amide, small ring) rotatable bonds (range for 95% of drugs: 0–15); ^h^ estimated number of hydrogen bonds that would be donated (don) by the solute to water molecules in an aqueous solution. Values are averages taken over a number of configurations, so they can be non-integer (range for 95% of drugs: 0.0–6.0); ^i^ estimated number of hydrogen bonds that would be accepted (acc) by the solute from water molecules in an aqueous solution. Values are averages taken over a number of configurations, so they can be non-integer (range for 95% of drugs: 2.0–20.0); ^j^ logarithm of partitioning coefficient between n-octanol and water (o/w) phases (range for 95% of drugs: −2–6.5); ^k^ logarithm of predicted aqueous (wat) solubility, logS. S in mol·dm^−3^ is the concentration of the solute in a saturated solution that is in equilibrium with the crystalline solid (range for 95% of drugs: −6.0–0.5); ^l^ logarithm of predicted binding constant to human serum albumin (HSA) (range for 95% of drugs: −1.5–1.5); ^m^ logarithm of predicted brain/blood partition coefficient. Note: QikProp predictions are for orally delivered drugs so, for example, dopamine and serotonin are CNS negative because they are too polar to cross the blood-brain barrier (range for 95% of drugs: −3.0–1.2); ^n^ predicted apparent Caco-2 cell membrane permeability in Boehringer-Ingelheim scale, in nm/s (range for 95% of drugs: <25 poor, > 500 great); ° number of likely metabolic reactions (range for 95% of drugs: 1–8); ^p^ predicted half-maximal inhibitory concentrations *IC*_50_^pre^. *IC*_50_^pre^ was predicted from computed ΔΔ*G*_com_ using the regression equation B shown in Table 3; ^q^ human oral absorption (1—low, 2—medium, 3—high); ^r^ percentage of human oral absorption in gastrointestinal tract (<25% poor; >80% high); * star indicating that the property descriptor value falls outside the range of values for 95% of known drugs.

## 3. Experimental Section

**Scheme 1:**
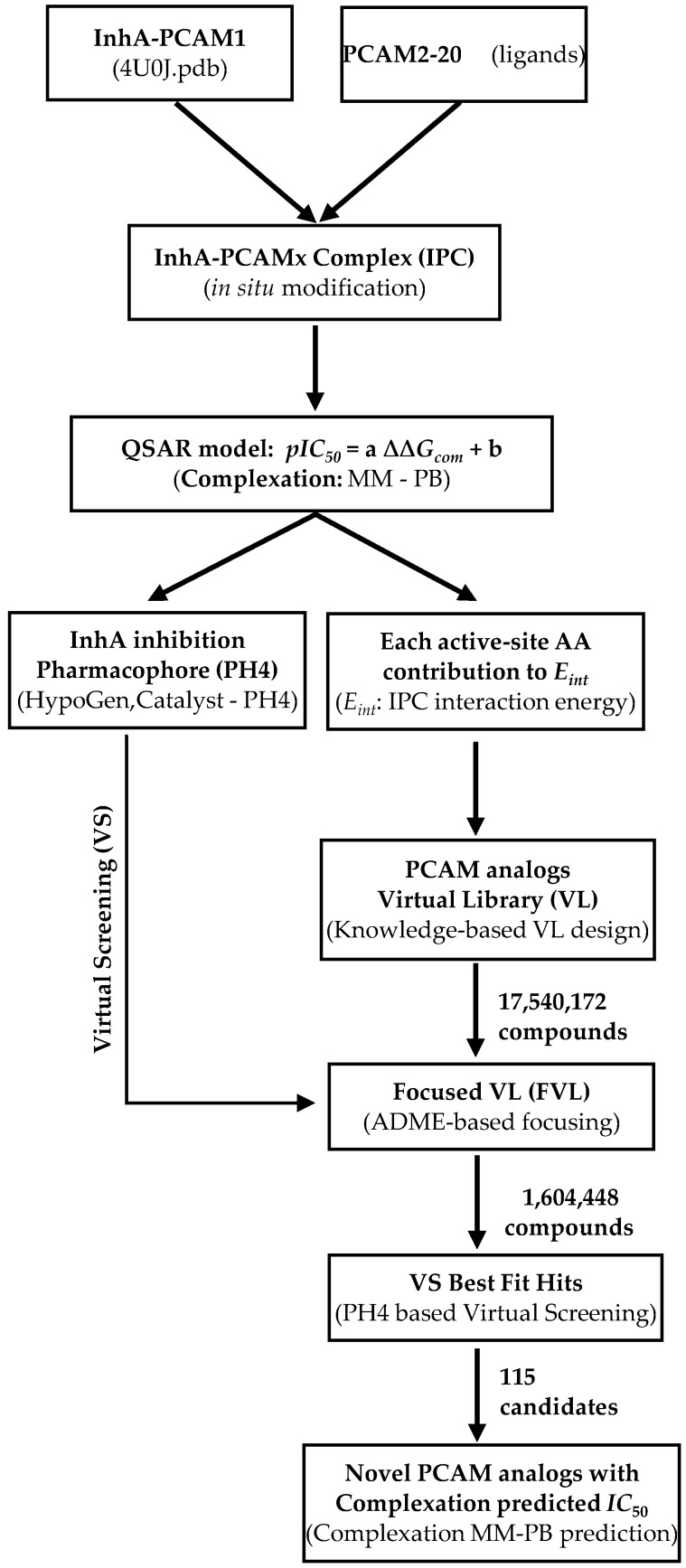
Workflow describing the multistep approach to virtually design novel PCAM analogues with higher predicted potency against InhA.

The workflow describing the steps of the whole process of virtual design of novel PCAM analogues is presented in Scheme 1.

### 3.1. Training and Validation Sets

Chemical structures and biological activities (*IC*_50_^exp^) of training and validation sets of pyrrolidine carboxamide inhibitors of InhA studied here were taken from literature [12]. The potencies of these compounds cover a broad range of half-maximal inhibitory concentrations (0.39 ≤ *IC*_50_^exp^ ≤ 101.0 μM) in order to allow construction of a QSAR model. The training set contained 20 PCAM inhibitors while the validation set included 3 PCAMs taken from the same reference [12].

### 3.2. Model Building

Three dimensional (3D) molecular models of enzyme-inhibitor complexes InhA-PCAMx, free enzyme InhA and free inhibitors PCAMx were constructed from high-resolution (1.62 Å) X-rays crystal structure of a reference complex composed of the training set compound 1-cyclohexyl-5-oxo-*N*-phenylpyrrolidine-3-carboxamide (PCAM1, Table 1) bound to the mycobacterial InhA (Protein Data Bank (PDB) [22] entry code 4U0J [12]) using Insight-II molecular modeling program [23].

The structures of InhA and enzyme-inhibitor (E:I) complexes were regarded to be at the pH of 7 with capped N- and C-terminal residues set to neutral charge. The protonizable and ionizable residues of the enzyme were considered charged. All crystallographic water molecules were removed from the model. The inhibitors were prepared from the reference crystal structure 4U0J [12] by *in situ* modification of function groups in the molecular scaffold of the reference inhibitor PCAM1. An exhaustive conformational search was carried out over all rotatable bonds of the replaced function groups, which was tied with a careful gradual energy-minimization of the inhibitor and active-site residues of the InhA located in the close vicinity (≤5 Å). This process helped to identify low-energy bound conformations of the modified inhibitors leading to stable structures of binary E:I complexes, which were then carefully refined by energy-minimization of the complex. This procedure has been successfully applied to building of models of viral, bacterial and protozoal enzyme-inhibitor complexes and design of peptidomimetic, hydroxynaphthoic, thymidine and triclosan-based enzyme inhibitors [16,24,25,26,27,28,29,30].

### 3.3. Molecular Mechanics

Modeling of inhibitors, InhA enzyme and E:I complexes was done in all-atom representation using consistent force field CFF91 force field parameters and charges [31]. In all molecular models a dielectric constant of 4 was employed for all molecular mechanics (MM) calculations in order to take into account the dielectric shielding effect in proteins. Energy minimizations of the E:I complexes, free E and I were completed by gradually relaxing the molecular structures, starting with the added hydrogen atoms, continued with heavy atoms of inhibitor, followed by residue side chains, and concluded with the protein backbone relaxation. During the geometry optimization process, a sufficient number of steepest descent steps followed by conjugate gradient iterative cycles were used, while the convergence criterion for the average gradient was set to 0.01 kcal·mol^−1^·Å^−1^.

### 3.4. Conformational Search

Conformations of free inhibitor were obtained from the bound conformations in the binary E:I complexes by gradual relaxation to the nearest local energy minimum. Then a Monte Carlo search (≤50,000 iterations) for low-energy conformations was performed over all rotatable bonds, except those in the rings, using Discovery Studio 2.5 (DS 2.5) molecular modeling program [32]. Two hundred unique inhibitor conformations were generated by randomly varying torsion angles of the last accepted conformer by ±15 deg at 5000 K followed by subsequent energy minimization. During the minimization a dielectric constant ε = 80 was used to approximate the dielectric screening effect of solvation. The conformer with the lowest total energy was selected and re-minimized at a dielectric constant of 4.

### 3.5. Solvation Gibbs free energies

The electrostatic component of the solvation Gibbs free energy which includes the effect of ionic strength via solving the nonlinear Poisson-Boltzmann equation [33,34] was computed by the DelPhi module of Discovery Studio [32]. The program represents the solvent by a continuous medium of high dielectric constant (ε_o_ = 80) and the solute as charge distribution filling a low dielectric (ε_i_ = 4) cavity with boundaries linked to the solute’s molecular surface. The program numerically solves for the molecular electrostatic potential and reaction field around the solute using finite difference method. DelPhi calculations were done on a (235 × 235 × 235) cubic lattice grid for the E:I complexes and free E and on (65 × 65 × 65) grid for the free I. Full coulombic boundary conditions were employed. Two subsequent focusing steps led to a similar final resolution of about 0.3 Å per grid unit at 70% filling of the grid by the solute. Physiological ionic strength of 0.145 mol·dm^−3^, atomic partial charges and radii defined in the CFF force field parameter set [32] and a probe sphere radius of 1.4 Å were used. The electrostatic component of the Poisson Boltzmann solvation Gibbs free energy was calculated as the reaction field energy [29,30,35,36,37].

### 3.6. Calculation of Binding Affinity and QSAR Model

The standard Gibbs free energy (GFE) change of enzyme-inhibitor (E:I) complexes (Δ*G*_com_) formation in water is related to inhibition constant (K_i_) of a reversible inhibitor I. Thus, prediction of the *K*_i_ value from computed complexation GFE as ln*K*_i_ = −Δ*G*_com_/R*T*, is achievable assuming the following equilibrium [25]:

{E}_aq_ + {I}_aq_ ↔ {E:I}_aq_ (1)

where {}_aq_ indicates solvated species. Half-maximal inhibitory concentration *IC*_50_ is for tight binding competitive inhibitors proportional to *K*_i_:

*IC*_50_ = *K*_i_·(*S*/*K*_m_ + 1) + *E*/2 (2)

where *S* is the substrate concentration, *K*_m_ represents the Michaelis constant and *E* means the free enzyme concentration [38]. The standard GFE change of the reaction Equation (1) can be estimated from molecular simulations of the complex and free reactants:

Δ*G*_com_ = *G*{E:I} − *G*{E} − *G*{I} (3)

In our laboratory we approximate the exact values of standard GFE for larger systems such as E:I complexes by expression [25,26,27]:

*G*{E:I} ≈ *E*_MM_{E:I} + *RT* − *TS*_trv_{E:I} + *G*_sol_{E:I} (4)

where *E*_MM_{E:I} stands for MM total energy of the complex (including bonding and non-bonding contributions), *G*_sol_{E:I} is the solvation GFE and *TS*_trv_{E:I} is the entropic term:

*TS*_trv_{E:I} = *TS*_tran_{E:I} + *TS*_rot_{E:I} + *TS*_vib_{E:I} (5)

composed of the sum of contributions arising from translational, rotational and vibrational motions of E:I. Assuming that the *tran* and *rot* terms for the complex E:I and free enzyme *E* are approximately equal, we obtain:

Δ*G*_com_ ≈ [*E*_MM_{E:I} − *E*_MM_{E} − *E*_MM_{I}] + [*G*_sol_{E:I} − *G*_sol_{E} − *G*_sol_{I}]
+ *T*S_tran_{I} + *TS*_rot_{I} − [*TS*_vib_{E:I} − *TS*_vib_{E} − *TS*_vib_{I}] = Δ*H*_MM_ + *TS*_tran_{I} + *TS*_rot_{I} − Δ*TS*_vib_ + Δ*G*_sol_ (6)

where *TS*_tran_{I} and *TS*_rot_{I} describe the translational and rotational entropy terms of the free inhibitor and Δ*TS*_vib_ represents a simplified vibrational entropy change upon the complex formation: Δ*TS*_vib_ = *TS*_vib_{I}_E_ − *TS*_vib_{I} [39,40]. Similarly, ΔΔ*H*_MM_ is the relative enthalpic contribution to the GFE change related to the intermolecular interactions in the E:I complex derived by MM.

Relative changes in the complexation GFE of different inhibitors with respect to a reference inhibitor, I_ref_, were computed assuming ideal gas behavior for the rotational and translational motions of the inhibitors [25]:

ΔΔ*G*_com_ = Δ*G*_com_(I) − Δ*G*_com_(I_ref_) = ΔΔ*H*_MM_ − ΔΔ*TS*_vib_ + ΔΔ*G*_sol_ (7)

This evaluation of relative changes is preferable as it may lead to partial cancellation of errors originating from the approximate nature of the MM method, solvent and entropic effects description.

Quantitative structure-activity relationships (QSAR), in which a linear relationship between the computed relative GFE of the InhA-PCAM complex formation ΔΔ*G*_com_ for the receptor and observed inhibitory potencies *IC*_50_^exp^ specific to *MTb*, is assumed according to Equations (1) and (2):

p*IC*_50_^exp^ = −log_10_*IC*_50_^exp^ = *a*·ΔΔ*G*_com_ + *b* (8)

The QSAR model was prepared by linear regression for the training set of PCAMs [12] using ΔΔ*G*_com_ quantities calculated from Equation (7), where *a* and *b* are regression coefficients. This QSAR model (termed here also as a target-specific scoring function) was then evaluated with help of validation set, not included into the training set, Section 3.1 and employed for prediction of inhibitory potencies (*IC*_50_^pre^) of newly designed and modeled PCAM analogues.

### 3.7. Interaction Energy

The MM interaction energy (*E*_int_) protocol available in DS 2.5 [32] computes the non-bonded interactions (van der Waals and electrostatic terms) between enzyme residues and the inhibitor. The calculations were performed using CFF forcefield [32] with a dielectric constant of 4. The breakdown of *E*_int_ into active-site residue contributions reveals the significance of individual interactions and permits a comparative analysis, which leads to identification of affinity enhancing as well as unfavorable PCAM substitutions.

The interaction energy diagram displaying active-site residue contribution to the *E*_int_ permits identification of residues with the highest contribution to the ligand binding and suggests the position and type of structural modifications, which can lead to improvement of binding affinity as exemplified in the design of thymine-like inhibitors of TMPK of *MTb* [16].

### 3.8. Pharmacophore Generation

Pharmacophore modeling assumes that a set of key structural features responsible for biological activity of the compound is recognized by the active site during receptor binding. In this work the pharmacophore was prepared by the 3D-QSAR pharmacophore protocol of Catalyst HypoGen algorithm [41] implemented in DS 2.5 [32]. Bound conformations of PCAM inhibitors taken from the refined E:I complexes were considered for the constructing of the pharmacophore building. The top scoring pharmacophore hypothesis was prepared in three stages: constructive, subtractive and optimization step, from a set of most active PCAMs inhibitors. The inactive compounds served for the definition of excluded volume. During the pharmacophore generation, five features available in the HypoGen algorithm were selected: hydrophobic aromatic (HYdAr), hydrophobic aliphatic (HYd), hydrogen-bond donor (HBD), hydrogen-bond acceptor (HBA), and ring aromatic (Ar) feature. Default values of the adjustable parameters were kept during the pharmacophore generation, except the uncertainty on the biological activity, which was reduced to 1.25 instead of 3. This adjustment modified the uncertainty interval of experimental activity from a wide span 〈IC_50_/3, 3·IC_50_〉 to a relatively narrow one 〈4·IC_50_/5, 5·IC_50_/4〉, due to accuracy and homogeneity of the measured activities originating from the same laboratory [12]. The top ten pharmacophores were generated with the number of missing features set to 0. Finally, the best pharmacophore model was selected. The PH4 pharmacophore model was then evaluated with help of the validation set of PCAM inhibitors [12], Section 3.1. Generally a PH4 model, as the one described here, can be used to estimate the pIC_50_ of new analogues on the basis of their mapping to the pharmacophore features. In this study, priority was given to the PH4 based screening of ADME focused VLs.

### 3.9. ADME Properties

Properties that determine the pharmacokinetics profile of a compound, besides octanol/water partitioning coefficient, aqueous solubility, blood/brain partition coefficient, Caco-2 cell permeability, serum protein binding, number of likely metabolic reactions and other eighteen descriptors related to adsorption, distribution, metabolism and excretion (ADME properties) of the inhibitors were computed by the QikProp program [21] based on the methods of Jorgensen [19,20,42]. According to those methods, experimental results of more than 710 compounds among which about 500 drugs and related heterocycles were correlated with computed physicochemical descriptors resulting in an accurate prediction of molecule’s pharmacokinetic profile. Drug likeness (#stars) is represented by the number of descriptors that exceed the range of values determined for 95% of known drugs out of 24 selected descriptors computed by the QikProp [21]. Drug-likeness was used as the global compound selection criterion related to ADME properties. The calculation of the selected ADME descriptors were performed from 3D structures of compounds considered. These descriptors were used to assess the pharmacokinetics profile of designed compounds and served also for the VL focusing (see Section 3.11).

### 3.10. Virtual Combinatorial Library Generation

The analogue model building was performed with Molecular Operating Environment (MOE) program [43]. The library of analogues was enumerated by attaching R-groups (fragments, building blocks) onto PCAM scaffold using the Quasar CombiDesign module of MOE [43]. Reagents and chemicals considered in this paper were selected from the directories of chemicals available from the commercial sources [18]. Each analogue was built as a neutral molecule in the MOE program [43], its molecular geometry was refined by MM optimization through smart minimizer of Discovery Studio [32] at high convergence criteria (threshold on energy difference of 10^−4^ kcal·mol^−1^ and root mean square deviation (RMSD) of 10^−5^ Å), dielectric constant of 4, using class II consistent force field CFF [31], as described in the Section 3.3.

### 3.11. ADME-Based Library Focusing

Twenty four pharmacokinetics-related molecular descriptors available in QikProp [21], which characterize a wide spectrum of molecular properties as described in Section 3.9, e.g., such as molecular mass, total solvent-accessible molecular surface, hydrophobic portion of the solvent-accessible molecular surface, total volume of molecule enclosed by solvent-accessible molecular surface, number of non-trivial non-hindered rotatable bonds, estimated number of hydrogen bonds that would be donated by the solute to water molecules in an aqueous solution, estimated number of hydrogen bonds that would be accepted by the solute from water molecules, logarithm of partitioning coefficient between *n*-octanol and water phases, logarithm of predicted aqueous solubility, logarithm of predicted binding constant to human serum albumin, logarithm of predicted brain/blood partition coefficient, apparent Caco-2 cell membrane permeability in Boehringer–Ingelheim scale, number of likely metabolic reactions, percentage of human oral absorption in gastrointestinal tract, *etc.* Optimum ranges of these 24 descriptors were defined in terms of upper and lower bounds according to QikProp [21]. Only compounds with predicted drug-likeness (#stars, Section 3.9) equal to zero, were retained in the focused VL of drug-like PCAM analogues.

### 3.12. Pharmacophore-Based Library Focusing

The pharmacophore model (PH4) described in Section 3.8 was derived from the bound conformations of PCAMs at the active-site of InhA. The enumerated and ADME-focused virtual library was further focused by using the ligand pharmacophore mapping protocol available of Discovery Studio [32]. Within this protocol, each generated conformer of the analogues was geometry optimized by means of the CFF forcefield for a maximum of 500 energy minimization steps and subsequently aligned and mapped to the PH4 model in order to select the top ranking overlaps. Twenty best-fitting inhibitor conformers were saved and clustered into 10 conformational families according to their mutual RMSD by Jarvis-Patrick complete linkage clustering method [44]. The best representative of each cluster was considered in the virtual screening of analogues. Only those analogues mapping to all four PH4 features were retained for the *in silico* screening.

### 3.13. In Silico Screening

The conformer with the best mapping on the PH4 pharmacophore in each cluster of the focused library subset was selected for *in silico* screening by the complexation QSAR model. The relative GFE of E:I complex formation in water ΔΔ*G*_com_ was computed for each selected new analogue and then used for prediction of InhA inhibitory potencies (*IC*_50_^pre^) of the focused virtual library of PCAM analogues by inserting this parameter into the target-specific scoring function, Equation (9). The scoring function, which is specific for the InhA receptor of *MTb*: p*IC*_50_^pre^[InhA] = *a*·ΔΔ*G*_com_ + *b*, was parameterized using the QSAR model described above, Section 3.6.

## 4. Conclusions

Structural information from the crystal structure of InhA-PCAM1 complex guided us during preparation of a reliable QSAR model of inhibition of the InhA of *MTb* by pyrrolidine carboxamide inhibitors, which correlated computed Gibbs free energies of complex formation with observed inhibitory potencies. In addition to this QSAR model, we have derived a PH4 pharmacophore model for PCAM inhibitors using a training set of 20 and validation set of 3 PCAMs with known inhibitory activities [12]. Analysis of interactions between the InhA and PCAMs in the enzyme active-site directed us in our effort to design an initial diversity virtual combinatorial library of new PCAM analogues with multiple substitutions on the benzene ring. A focused library filtered by a set of ADME-related descriptors and screened by matching of the analogues to the PH4 pharmacophore, permitted selection of a library subset of orally bioavailable PCAMs. This subset of 115 best virtual hits was submitted to computation of predicted InhA inhibitory potencies by the complexation QSAR model. The best analogues reached predicted activities in the low nanomolar concentration range. The best designed PCAM analogues 6-2-1-4-7 (*IC*_50_^pre^ = 10.5 nM), 11-2-H-5-4 (*IC*_50_^pre^ = 8.6 nM), 6-2-H-4-7 (*IC*_50_^pre^ = 7.7 nM), 21-2-1-6-4 (*IC*_50_^pre^ = 7.6 nM), 21-2-H-6-4 (*IC*_50_^pre^ = 4.5 nM), and 4-2-H-6-5 (*IC*_50_^pre^ = 7.3 nM) Table 6, are recommended for synthesis and subsequent activity evaluation in InhA inhibition assays and may lead to a discovery of novel potent orally bioavailable antituberculotics.

## Supplementary Files

Supplementary File 1
