# Supplementary figures and images for: Computer-Aided Design of Orally Bioavailable Pyrrolidine Carboxamide Inhibitors of Enoyl-Acyl Carrier Protein Reductase of Mycobacterium tuberculosis with Favorable Pharmacokinetic Profiles
Source: Int J Mol Sci. 2015 Dec 12;16(12):29744–71. doi: 10.3390/ijms161226196 (PMC4691139; doi:10.3390/ijms161226196)

## Slide 1
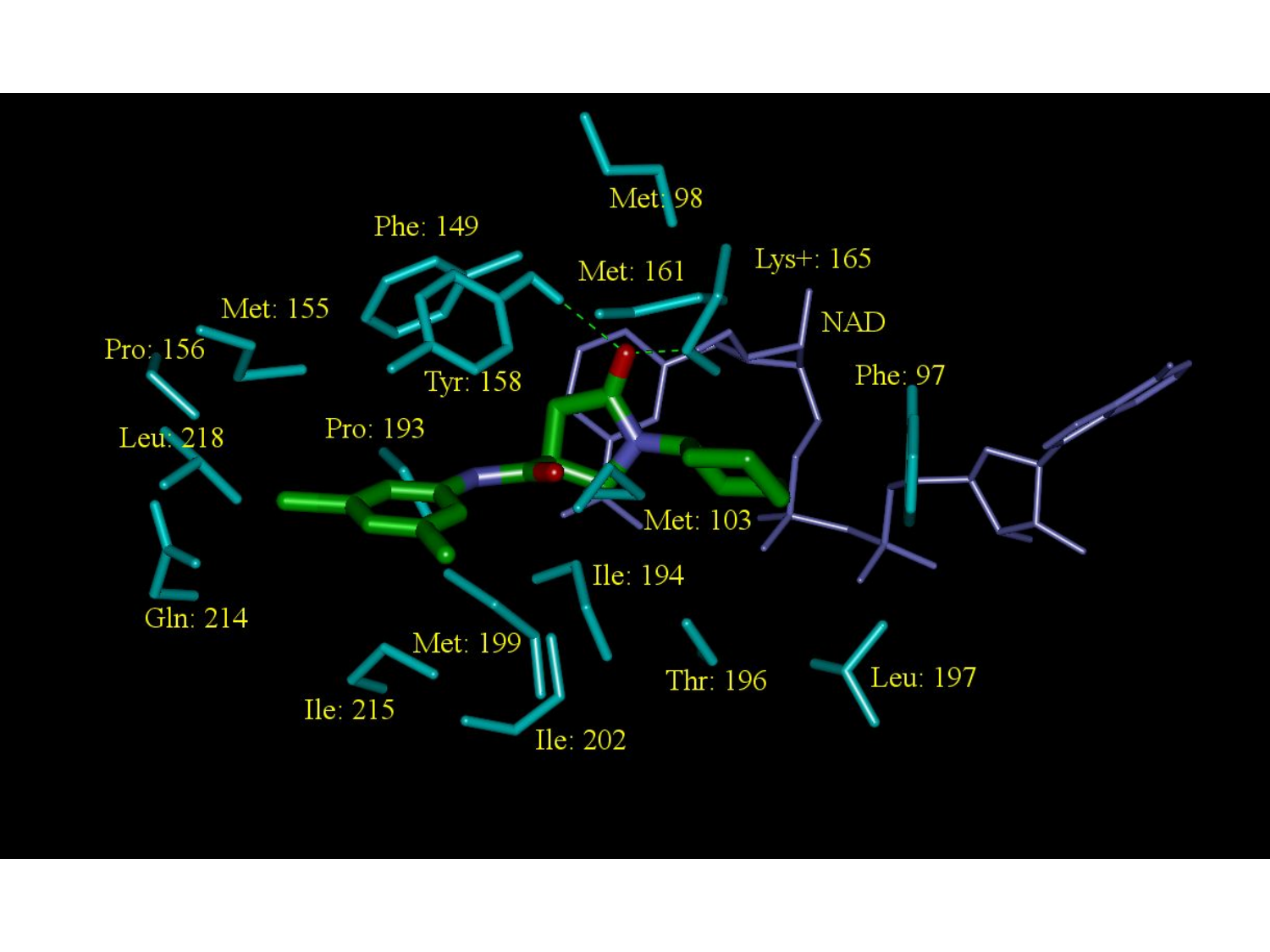

#

## Slide 2
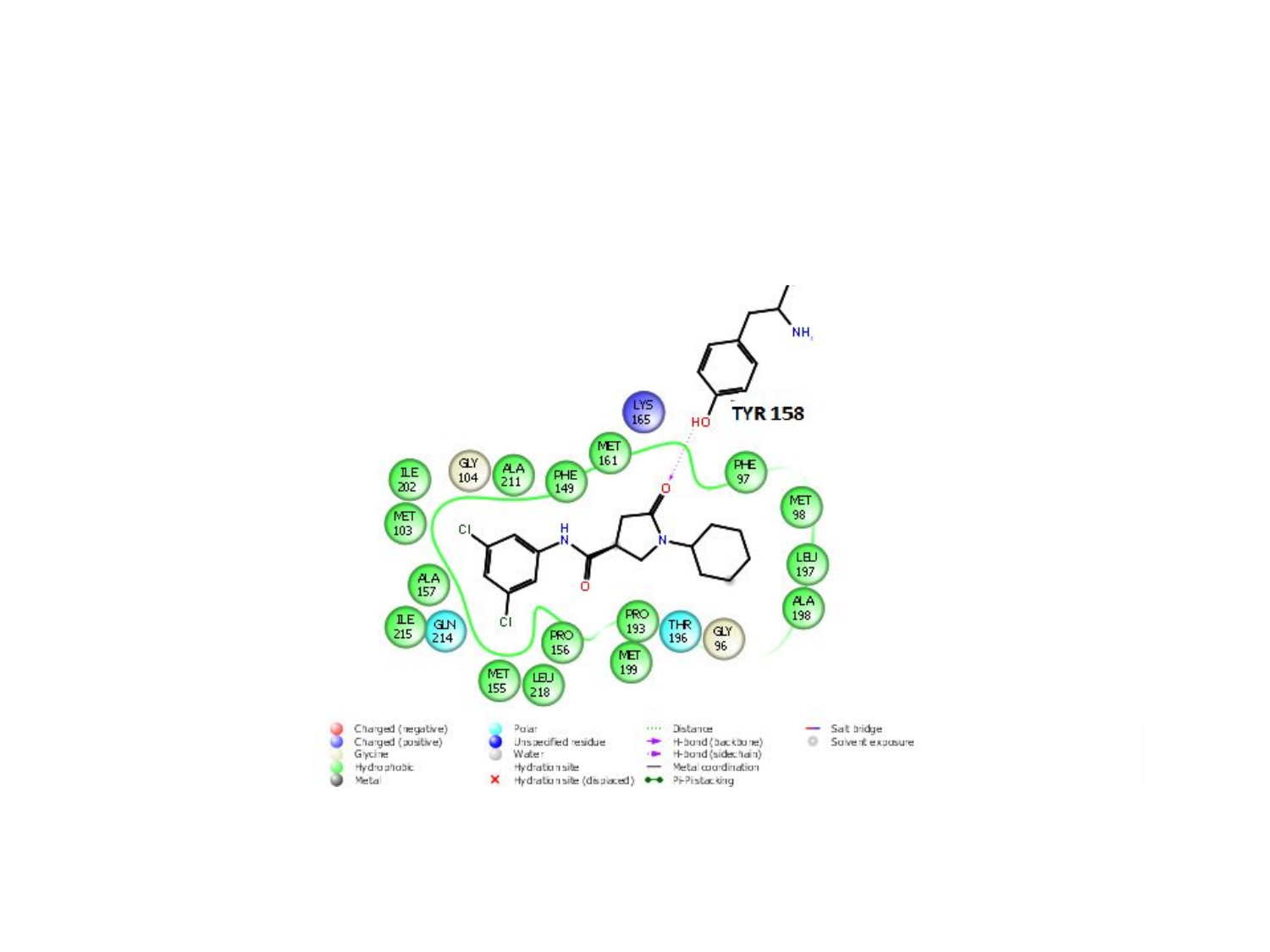

Supplement: Supplementary File 1 [file ijms-16-26196-s001.pptx]
